# Numerically Exploring the Potential of Abating the Energy Flow Peaks through Tough, Single Network Hydrogel Vibration Isolators with Chemical and Physical Cross-Links †

**DOI:** 10.3390/ma14040886

**Published:** 2021-02-13

**Authors:** Leif Kari

**Affiliations:** The Marcus Wallenberg Laboratory for Sound and Vibration Research (MWL), Department of Engineering Mechanics, KTH Royal Institute of Technology, 100 44 Stockholm, Sweden; leifkari@kth.se; Tel.: +46-70-798-7974

**Keywords:** vibration isolation system, rigid body resonance, single polymer network hydrogel, chemical cross-link, physical cross-link, high loss factor, adhesion–deadhesion activity, simulation model, energy flow reduction, polyvinyl alcohol hydrogel

## Abstract

Traditional vibration isolation systems, using natural rubber vibration isolators, display large peaks for the energy flow from the machine source and into the receiving foundation, at the unavoidable rigid body resonance frequencies. However, tough, doubly cross-linked, single polymer network hydrogels, with both chemical and physical cross-links, show a high loss factor over a specific frequency range, due to the intensive adhesion–deadhesion activities of the physical cross-links. In this study, vibration isolators, made of this tough hydrogel, are theoretically applied in a realistic vibration isolation system, displaying several rigid body resonances and various energy flow transmission paths. A simulation model is developed, that includes a suitable stress–strain model, and shows a significant reduction of the energy flow peaks. In particular, the reduction is more than 30 times, as compared to the corresponding results using the natural rubber. Finally, it is shown that a significant reduction is possible, also without any optimization of the frequency for the maximum physical loss modulus. This is a clear advantage for polyvinyl alcohol hydrogels, that are somewhat missing the possibility to alter the frequency for the maximum physical loss, due to the physical cross-link system involved—namely, that of the borate esterification.

## 1. Introduction

Noise, vibration and harshness are key quality discriminators while selecting a product to acquire—for example, cars, trucks, boats, air vessels and household appliances. Their impacts are generally even wider than that—for example, the consequences of airplane vibrations range from negligible through significant passenger disturbances to essential flight safety concerns. The vibrations are generated at the source and transmitted further via the surrounding structures, and are eventually radiating as noise. Both the vibration and the noise are usually perceived as disturbing by the human observers, and thus result in the subjective impression as unwanted. A simple and effective measure to reduce the vibrations and noise is to disconnect the source from the receiving structure by vibration isolators, thereby creating a mechanical mismatch, as vibration isolators are mechanically soft while the source and receiving structure are in contrary mechanically hard. The sudden mechanical mismatch reflects the incoming vibrations back to the source—thus, it is reducing the transmitted vibrations into the receiving structure and, thereby, is generally reducing the noise radiated. The inclusion of the damping in the vibration isolators, an inherent property of such as rubber, diminishes the vibrations by transforming them into heat at the inevitable rigid body resonances, created by the vibration isolation system while introducing more degrees-of-freedom to the system [1,2,3]. In practice, it is generally unavoidable to excite some of those rigid body resonances in a real vibration isolation system. Consequently, the damping in the vibration isolators should be at its maximum at those frequencies to avoid excess motion amplitudes. On the contrary, the damping in the vibration isolators should not be excessive at the higher frequencies, well above the rigid body resonances, as the vibration isolation actually decreases with increased damping at the higher frequencies, except for the frequencies at and close to the internal anti-resonances of the vibration isolators, where some damping is necessary [1,2,3]. In conclusion, the damping in the vibration isolators should be at its maximum in the low-frequency, rigid body resonance range of the vibration isolation system, while being substantially smaller, although non-vanishing, at the higher frequencies. Engineering materials applied in the vibration isolators, such as natural rubber, typically display a low-to-moderate damping in the low-frequency range and is slightly, to moderately, increasing with growing frequency. That is, the typical engineering materials applied in vibration isolators are not meeting the optimal frequency characteristic of their damping. However, tough hydrogels, essentially consisting of diverse cross-linked network configurations of macromolecules, while containing hydrophilic functional groups and being profusely swollen with water, are an interesting and possible vibration isolator material [4,5], although they are typically up to now applied in tissue engineering. It should be noted that there are also other possible vibration isolator materials, including high damping visco-elastic materials [6], auxetic cellular materials [7] and thermo-plastic elastomers [8]. However, the tough hydrogel studied in this paper most likely meets the optimal damping frequency characteristic—displaying a maximum loss factor at the rigid body resonances while being substantially lower sufficiently outside that frequency range.

In this paper, plausible vibration isolators made of tough, doubly cross-linked, single network hydrogels, concurrently accommodating both chemical and physical cross-links [9,10,11,12,13,14,15,16,17,18,19,20,21,22,23,24,25,26,27,28,29,30,31,32,33,34], are theoretically applied in a more realistic vibration isolation system, as compared to that in the preliminary investigation published at the Medyna 2020 conference [5] and to the experimental study in Yang et al. [4], the latter using tough hydrogels with various multivalent cations. In general, the chemical cross-links consist of covalent bonds, while the physical cross-links may include hydrogen bonds, van der Waals, hydrophobic, π – π and ionic; ion–ion, ion–dipole, dipole–dipole interactions, see for example Refs. [17,35,36,37,38]. The tough hydrogel generally shows an adjustable maximum physical shear loss modulus frequency and adjustable low and high frequency shear storage modula [24]. The adjustability is a promising property and well worth to investigate whether this plausible material for the vibration isolators meets the previous desired frequency characteristic of its damping, in contrast to the natural rubber. The specific tough hydrogel studied is a doubly cross-linked polyvinyl alcohol, for which constitutive models has been derived, see for example Refs. [12,15,16,18,28,31,33]. Those constitutive models embrace finite strains and fractional time power dependencies [12], generalized Stokes–Einstein equation [15], survivability functions [16,18,28,31] and fractional time derivatives [33]. The latter model applies only four physically comprehensible material parameters, while still showing an associative Rouse mode low-frequency response [39]—that is, a shear modulus with a low-frequency dependence of order 1/2, in addition to a frequency independent part. Furthermore, the model [33] makes it possible to additively divide into the contributions from the chemical and the physical cross-links, respectively. It displays results close to those of the measurements and is the constitutive model applied in this paper. Visco-elastic fractional time derivative models are regularly applied, see for example Refs. [40,41,42,43,44,45,46,47,48,49,50,51,52,53,54,55,56,57,58,59,60,61,62,63,64,65,66,67,68,69,70,71,72,73,74,75,76,77,78,79,80,81,82,83,84,85,86,87,88,89,90]. Moreover, fractional time derivatives are, in addition, used for modeling chemical and physical aging of rubber [91,92], and in more areas, as reviewed by Machado et al. [93]. Although, the constitutive model applied in the energy flow modeling is previously fitted to the measurement results of the polyvinyl alcohol hydrogels [33], the energy modeling results are, nevertheless, applicable for a wider class of tough, doubly cross-linked, single network hydrogels with chemical and physical cross-links, provided that similar stress–strain relations are relevant.

Cylindrical bushings are frequently applied as vibration isolators, due to their axial and torsional flexibility, in combination with their radial and tilt rigidity. Examples of static stiffness models of the cylindrical bushings include finite element models [94,95,96,97,98], principal mode models [99], truncated Fourier and Bessel function models [100] and analytical models [101,102]. Likewise, examples of dynamic stiffness models include finite element models [64,103,104], combination of finite element and empirical models [105], analytical models [52,69], waveguide models [59] and equivalent strains and analytical models [60,62,63]. However, none of the stiffness model investigations mentioned above have applied tough hydrogel, instead of rubber in their formulas. The simple, straightforward and analytical, axial dynamic stiffness model for long rubber bushings [52], is applied in this paper, with the rubber replaced with the tough hydrogel. This model has, furthermore, shown to give the axial stiffness results close to those of the more accurate, yet more complex and time consuming models—such as the waveguide models [59], for sufficiently long bushings.

A straightforward and constructive measure to evaluate the vibration isolation achieved, is to determine the energy flow transmitted through the vibration isolators into the receiving structure, as it simultaneously includes both the forces and velocities transmitted, and compare it to the case without the vibration isolators. An early account on the energy flow through the vibration isolators was performed by Lyon and Maidanik [106], already in 1962, analyzing the energy (power) flow through a two-stage vibration isolation system, by Goyder and White, in a series of papers [107,108,109], investigating the vibration isolation of machines and the energy flow (power transmission) processes in sub-structures, and by Pinnington and White [110], and Pinnington [111,112], in a series of papers, investigating the energy (power) flow through the vibration isolators into a seating. Subsequently, a number of studies have been conducted, including the analysis of the energy (power) flow from a machine through multiple vibration isolators and into a supporting structure [113], and into a cylindrical shell [114], and studies of the energy (power) flow through multi-dimensional vibration isolation systems [115]. They also include studies of the energy (power) flow through vibration isolators during an earthquake [116], through vibration isolators into a floating panel [117], through magneto-sensitive vibration isolators [71,85,118,119,120], through non-linear vibration isolators [121,122] and through steel springs with distributed mass [123]. Furthermore, they include studies of the energy (power) flow from a centrifugal turbo blower into a chassis frame [124], in a two-stage non-linear vibration isolation system [125] and in a two-stage inerter-based vibration isolation system [126]. Finally, they include calculations and in situ measurements of the energy (power) flow transmitted through vibration isolators to a seating structure [127,128], in situ measurements of the energy (power) flow through elastomeric powertrain vibration isolators in a passenger car [129] and investigations of the energy (power) flow transmissibility as a measure to evaluate the capacity of an isolation system [130]. However, none of the energy (power) flow investigations, mentioned above, have applied the tough hydrogel vibration isolators. A literature review on the energy flow studies through interfaces between interacting structures, is found in Acri [131].

Previously, it has been concluded that this tough hydrogel is a plausible material in the dynamic vibration absorber springs [34]; in particular, while selecting a not too small dynamic vibration absorber mass to elude an excess displacement amplitude of the dynamic vibration absorber spring, where a high loss factor is required [34]. It is now time to study a more common vibration reduction measure—namely, its suitability in vibration isolation systems, in particular a more realistic, multi-degree system, as compared to the initial experimental, one-dimensional study by Yang [4]—the latter using various multivalent cations. Although dynamic vibration absorbers and vibration isolation find their applications in the vibration abatement area, they are, nevertheless, poles apart. Dynamic vibration absorbers work with counter forces, at the desired frequencies, to reduce the vibrations of the primary vibration system they are attached to, where the damping is included to increase their effective bandwidth, to dissipate mechanical energy and to reduce their sensitivity to design parameter deviations. On the contrary, vibration isolation work with mechanical mismatches, to reflect the incoming vibrations back to the source and where the damping is included to convert mechanical energy into heat, at the rigid body resonances of the vibration isolation system in the low-frequency range and at the internal resonances/anti-resonances within the vibration isolator spring in the high-frequency range.

This paper extends the simple, single torsional vibration isolation system presented at the Medyna 2020 conference [5], showing a single rigid body resonance, into a more realistic vibration isolation system, with a solid rectangular block excited by a force and a moment, as the mechanical source, and connected to an infinite foundation, via four tough, single network hydrogel vibration isolator bushings, simultaneously embodying both chemical and physical cross-links. This realistic system is displaying several rigid body resonances and various transmission paths for the energy flow. The numerical investigation carried out in this paper reveals whether it is possible to achieve an increased reduction of the energy flow, through the tough hydrogel vibration isolator bushings and to what extent, in particular at the rigid body resonance frequencies, as compared to the corresponding results while using the more traditional rubber vibration isolators.

## 2. Materials and Methods

**Figure 1:**
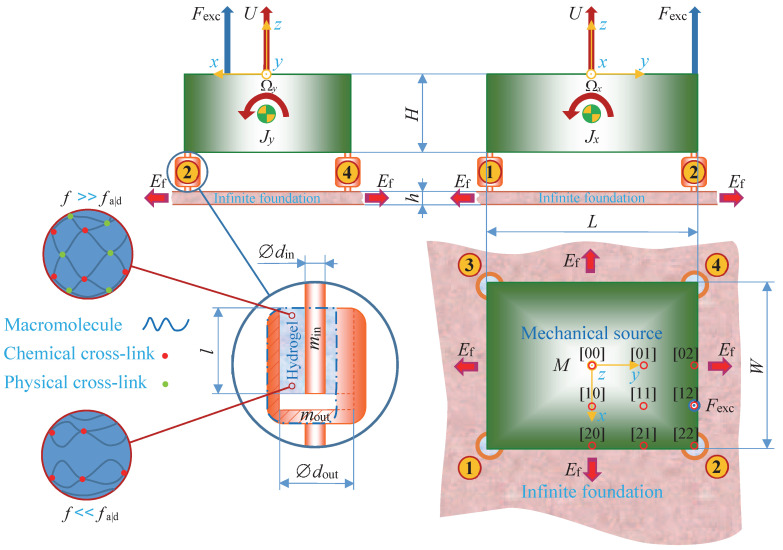
A mechanical source consisting of a solid metal block and excited by a force at a point [n m], n,m=0,1,2. The mechanical source is connected to an infinite metal foundation via four identical vibration isolator bushings, at its lower surface corners. The vibration isolator bushings consist of a tough, single network hydrogel, with both chemical and physical cross-links and are bonded between an inner and outer metal sleeve. First-angle projection applied.

The mechanical source, in Figure 1, consists of a solid metal block of the dimensions W×L×H, the density $\rho_{M}$, the mass $M=\rho_{M}WLH$, the moments of inertia $J_{x}=M\left(L^{2}+H^{2}\right)/12$ and $J_{y}=M\left(W^{2}+H^{2}\right)/12$, around the *x*- and *y*-axis, respectively, and is excited by a force $F_{\mathrm{exc}}\left(t\right)$ parallel to the *z*-axis, on a point at its upper surface, where *t* is the time. Its rigid body motion is given in full by the generalized displacement vector $u\left(t\right)={\left(U\left(t\right),W_{x}\left(t\right),W_{y}\left(t\right)\right)}^{T}$, where U(t) is the rectilinear displacement, parallel to the *z*-axis, at the center point on the upper surface, $W_{x}\left(t\right)$ and $W_{y}\left(t\right)$ are the rotational displacement around the *x*- and *y*-axis, respectively, at the center of gravity, and T denotes the transpose. The time dependent variables are transformed into the corresponding frequency dependent variables through the temporal Fourier transformation $\left(\tilde{\cdot }\right)=\int_{-\infty }^{+\infty }\left(\cdot \right)\exp\left(-i\omega t\right)d\;t$, where i is the imaginary unit, ω=2πf is the angular frequency and *f* is the frequency. The mechanical source is connected to an infinite foundation of the density $\rho_{f}$, the Young’s modulus $Y_{f}$, the Poisson’s ratio $\nu_{f}$ and of the thickness *h*, via four identical vibration isolator bushings, at its lower surface corners.

**Figure 2:**
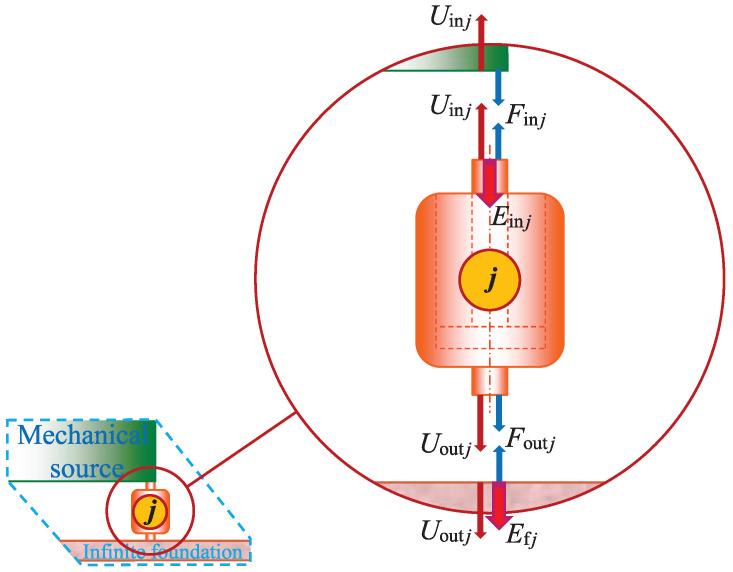
Displacements, forces and time averaged energy flows at the connecting surfaces to the mechanical source and to the infinite metal foundation for the vibration isolator bushing ⓙ, where j=1,2,3,4.

The vibration isolator bushings, in Figure 1, consist of the tough, single network hydrogel, with chemical and physical cross-links, of the length *l* and are bonded between the diameters $d_{\mathrm{in}}$ and $d_{\mathrm{out}}$, to inner and outer metal sleeves of the mass $m_{\mathrm{in}}$ and $m_{\mathrm{out}}$, respectively. The axial dynamic stiffness components are the axial inner dynamic driving point stiffness

(1) $$K_{\mathrm{in}\;\mathrm{in}}=2\pi r_{\mathrm{in}}lk_{T}\mu \frac{J_{1}\left(k_{T}r_{\mathrm{in}}\right)Y_{0}\left(k_{T}r_{\mathrm{out}}\right)-J_{0}\left(k_{T}r_{\mathrm{out}}\right)Y_{1}\left(k_{T}r_{\mathrm{in}}\right)}{J_{0}\left(k_{T}r_{\mathrm{in}}\right)Y_{0}\left(k_{T}r_{\mathrm{out}}\right)-J_{0}\left(k_{T}r_{\mathrm{out}}\right)Y_{0}\left(k_{T}r_{\mathrm{in}}\right)}-\omega^{2}m_{\mathrm{in}},$$

the axial dynamic outer driving point stiffness

(2) $$K_{\mathrm{out}\;\mathrm{out}}=2\pi r_{\mathrm{out}}lk_{T}\mu \frac{J_{1}\left(k_{T}r_{\mathrm{out}}\right)Y_{0}\left(k_{T}r_{\mathrm{in}}\right)-J_{0}\left(k_{T}r_{\mathrm{in}}\right)Y_{1}\left(k_{T}r_{\mathrm{out}}\right)}{J_{0}\left(k_{T}r_{\mathrm{in}}\right)Y_{0}\left(k_{T}r_{\mathrm{out}}\right)-J_{0}\left(k_{T}r_{\mathrm{out}}\right)Y_{0}\left(k_{T}r_{\mathrm{in}}\right)}-\omega^{2}m_{\mathrm{out}}$$

and the axial dynamic transfer stiffness

(3) $$K_{\mathrm{in}\;\mathrm{out}}=K_{\mathrm{out}\;\mathrm{in}}=\frac{4l\mu }{J_{0}\left(k_{T}r_{\mathrm{in}}\right)Y_{0}\left(k_{T}r_{\mathrm{out}}\right)-J_{0}\left(k_{T}r_{\mathrm{out}}\right)Y_{0}\left(k_{T}r_{\mathrm{in}}\right)},$$

for sufficiently long bushings ($l\gg d_{\mathrm{out}}-d_{\mathrm{in}}$) [52], where the inner radius $r_{\mathrm{in}}=d_{\mathrm{in}}/2$, the outer radius $r_{\mathrm{out}}=d_{\mathrm{out}}/2$, the Bessel functions of first and second kind and of order *p* are $J_{p}$ and $Y_{p}$, respectively. Moreover, the transversal wavenumber    

(4) $$k_{T}=\omega \sqrt{\frac{\rho }{\mu }},$$

the hydrogel density is ρ, the hydrogel shear modulus

(5) $$\mu =\underset{\mu_{\mathrm{chem}}}{\underbrace{\mu_{\mathrm{st}}\left[1+C\sqrt{i\omega }\right]}}+\underset{\mu_{\mathrm{phys}}}{\underbrace{\mu_{\mathrm{st}}\frac{\Delta \sqrt{i\frac{\omega }{\omega_{a|d}}}}{1+\sqrt{i\frac{\omega }{\omega_{a|d}}}}}},$$

according to the 4-parameters model in Kari [33], with an additive split into a chemical shear modulus part $\mu_{\mathrm{chem}}$ and a corresponding physical part $\mu_{\mathrm{phys}}$, the static shear modulus $\mu_{\mathrm{st}}=\lim_{\omega \to 0}\mu \left(\omega \right)$, the angular frequency for the maximum (physical) loss shear modulus is $\omega_{a|d}$ (assuming C=0), the non-dimensional relaxation intensity $\Delta =\lim_{\omega \to \infty }\left[\mu \left(\omega \right)/\mu_{\mathrm{st}}\right]-1$ (assuming C=0) and the chemical Rouse stress intensity factor $C\ll 1\sqrt{s}$. Consequently, the relations between the vibration isolator forces and displacements, in Figure 2, are

(6) $${\tilde{f}}_{\mathrm{in}}=K_{\mathrm{in}\;\mathrm{in}}{\tilde{u}}_{\mathrm{in}}+K_{\mathrm{in}\;\mathrm{out}}{\tilde{u}}_{\mathrm{out}}$$

and

(7) $${\tilde{f}}_{\mathrm{out}}=K_{\mathrm{out}\;\mathrm{in}}{\tilde{u}}_{\mathrm{in}}+K_{\mathrm{out}\;\mathrm{out}}{\tilde{u}}_{\mathrm{out}},$$

where ${\tilde{f}}_{\mathrm{in}}={\left({\tilde{F}}_{\mathrm{in}\;1},{\tilde{F}}_{\mathrm{in}\;2},{\tilde{F}}_{\mathrm{in}\;3},{\tilde{F}}_{\mathrm{in}\;4}\right)}^{T}$, ${\tilde{u}}_{\mathrm{in}}={\left({\tilde{U}}_{\mathrm{in}\;1},{\tilde{U}}_{\mathrm{in}\;2},{\tilde{U}}_{\mathrm{in}\;3},{\tilde{U}}_{\mathrm{in}\;4}\right)}^{T}$, ${\tilde{f}}_{\mathrm{out}}=({\tilde{F}}_{\mathrm{out}\;1},{\tilde{F}}_{\mathrm{out}\;2},$${\tilde{F}}_{\mathrm{out}\;3},{\tilde{F}}_{\mathrm{out}\;4}{)}^{T}$ and ${\tilde{u}}_{\mathrm{out}}={\left({\tilde{U}}_{\mathrm{out}\;1},{\tilde{U}}_{\mathrm{out}\;2},{\tilde{U}}_{\mathrm{out}\;3},{\tilde{U}}_{\mathrm{out}\;4}\right)}^{T}\;$. Kinematically, the relation between the isolator displacement vector, at the machine source connection points, and the mechanical source displacement vector is ${\tilde{u}}_{\mathrm{in}}=A\tilde{u}$, where the kinematic (4×3) matrix reads

(8) $$A=\left[\begin{array}{ccc} 1 & -\frac{L}{2}+r_{\mathrm{in}} & -\frac{W}{2}+r_{\mathrm{in}}\\ 1 & +\frac{L}{2}-r_{\mathrm{in}} & -\frac{W}{2}+r_{\mathrm{in}}\\ 1 & -\frac{L}{2}+r_{\mathrm{in}} & +\frac{W}{2}-r_{\mathrm{in}}\\ 1 & +\frac{L}{2}-r_{\mathrm{in}} & +\frac{W}{2}-r_{\mathrm{in}} \end{array}\right]$$

and the inner metal sleeves of the vibration isolator bushings are neatly attached to the machine source, at its lower surface corners, see Figure 1 and Figure 2.

The compliance relation between the vibration isolator displacement vector, at the foundation connection points, and the corresponding force vector is ${\tilde{u}}_{\mathrm{out}}=-H{\tilde{f}}_{\mathrm{out}}$, where the compliance (4×4) matrix reads

(9) $$\begin{array}{cc} H & =\frac{1}{8iBk_{f}^{2}}\\ \; & \;\times \left[\begin{array}{cccc} 1 & \;\prod (k_{f}\left[L-2r_{\mathrm{in}}\right]) & \;\prod (k_{f}\left[W-2r_{\mathrm{in}}\right]) & \;\prod \left(k_{f}\sqrt{{\left[W-2r_{\mathrm{in}}\right]}^{2}+{\left[L-2r_{\mathrm{in}}\right]}^{2}}\right)\\ \prod (k_{f}\left[L-2r_{\mathrm{in}}\right]) & \;1 & \;\prod \left(k_{f}\sqrt{{\left[W-2r_{\mathrm{in}}\right]}^{2}+{\left[L-2r_{\mathrm{in}}\right]}^{2}}\right) & \;\prod (k_{f}\left[W-2r_{\mathrm{in}}\right])\\ \prod (k_{f}\left[W-2r_{\mathrm{in}}\right]) & \;\prod \left(k_{f}\sqrt{{\left[W-2r_{\mathrm{in}}\right]}^{2}+{\left[L-2r_{\mathrm{in}}\right]}^{2}}\right) & \;1 & \;\prod (k_{f}\left[L-2r_{\mathrm{in}}\right])\\ \prod \left(k_{f}\sqrt{{\left[W-2r_{\mathrm{in}}\right]}^{2}+{\left[L-2r_{\mathrm{in}}\right]}^{2}}\right) & \;\prod (k_{f}\left[W-2r_{\mathrm{in}}\right]) & \;\prod (k_{f}\left[L-2r_{\mathrm{in}}\right]) & \;1 \end{array}\right], \end{array}$$

where the bending stiffness of the foundation $B=Y_{f}h^{3}/12\left(1-\nu_{f}^{2}\right)$, the bending wavenumber of the foundation $k_{f}={\left(\rho_{f}h\omega^{2}/B\right)}^{1/4}$, $\prod \left(x\right)=H_{0}^{\left(2\right)}\left(x\right)-2iK_{0}\left(x\right)/\pi$, the Hankel function of second kind and zero order is $H_{0}^{\left(2\right)}$ and the modified Bessel function of second kind and zero order is $K_{0}$ [85,132].

The excitation force $f_{\mathrm{exc}}$ is possible to apply to several points [n m] at the upper surface of the machine source, according to Figure 1, where the excitation point coordinates are given by $\left[x_{\mathrm{exc}},y_{\mathrm{exc}}\right]=\left[n\left\{\left(W/2\right)-r_{\mathrm{in}}\right\}/2,m\left\{\left(L/2\right)-r_{\mathrm{in}}\right\}/2\right]$ and n,m=0,1,2. Newton’s second law, applied to the motion of the machine source, reads $-\omega^{2}M\tilde{u}=-B{\tilde{f}}_{\mathrm{in}}+c{\tilde{f}}_{\mathrm{exc}}$, where the generalized mass (3×3) matrix $M=\mathrm{diag}(M,J_{x},J_{y})$, the (3×4) matrix

(10) $$B=\left[\begin{array}{cccc} 1 & 1 & 1 & 1\\ -\frac{1}{2}\left(L-2r_{\mathrm{in}}\right) & +\frac{1}{2}\left(L-2r_{\mathrm{in}}\right) & -\frac{1}{2}\left(L-2r_{\mathrm{in}}\right) & +\frac{1}{2}\left(L-2r_{\mathrm{in}}\right)\\ -\frac{1}{2}\left(W-2r_{\mathrm{in}}\right) & -\frac{1}{2}\left(W-2r_{\mathrm{in}}\right) & +\frac{1}{2}\left(W-2r_{\mathrm{in}}\right) & +\frac{1}{2}\left(W-2r_{\mathrm{in}}\right) \end{array}\right]$$

and the vector

(11) $$c=\left(\begin{array}{c} 1\\ +\frac{m}{4}\left(L-2r_{\mathrm{in}}\right)\\ -\frac{n}{4}\left(W-2r_{\mathrm{in}}\right) \end{array}\right).$$

The resulting vibration isolator displacement vector at the foundation reads  

(12) $${\tilde{u}}_{\mathrm{out}}=-K_{\mathrm{out}\;\mathrm{in}}{\left(1+K_{\mathrm{out}\;\mathrm{out}}H\right)}^{-1}HA{\left[K_{\mathrm{in}\;\mathrm{in}}BA-K_{\mathrm{out}\;\mathrm{in}}^{2}B{\left(1+K_{\mathrm{out}\;\mathrm{out}}H\right)}^{-1}HA-\omega^{2}M\right]}^{-1}c{\tilde{f}}_{\mathrm{exc}}$$

and the corresponding force vector reads  

(13) $${\tilde{f}}_{\mathrm{out}}=K_{\mathrm{out}\;\mathrm{in}}{\left(1+K_{\mathrm{out}\;\mathrm{out}}H\right)}^{-1}A{\left[K_{\mathrm{in}\;\mathrm{in}}BA-K_{\mathrm{out}\;\mathrm{in}}^{2}B{\left(1+K_{\mathrm{out}\;\mathrm{out}}H\right)}^{-1}HA-\omega^{2}M\right]}^{-1}c{\tilde{f}}_{\mathrm{exc}},$$

where the unit (4×4) matrix 1=diag(1,1,1,1). Likewise, the resulting vibration isolator displacement vector at the machine source reads

(14) $${\tilde{u}}_{\mathrm{in}}=A{\left[K_{\mathrm{in}\;\mathrm{in}}BA-K_{\mathrm{out}\;\mathrm{in}}^{2}B{\left(1+K_{\mathrm{out}\;\mathrm{out}}H\right)}^{-1}HA-\omega^{2}M\right]}^{-1}c{\tilde{f}}_{\mathrm{exc}}$$

and the corresponding force vector reads

(15) $$\begin{array}{cc} {\tilde{f}}_{\mathrm{in}}= & \left[K_{\mathrm{in}\;\mathrm{in}}A-K_{\mathrm{out}\;\mathrm{in}}^{2}{\left(1+K_{\mathrm{out}\;\mathrm{out}}H\right)}^{-1}HA\right]\\ \; & \times {\left[K_{\mathrm{in}\;\mathrm{in}}BA-K_{\mathrm{out}\;\mathrm{in}}^{2}B{\left(1+K_{\mathrm{out}\;\mathrm{out}}H\right)}^{-1}HA-\omega^{2}M\right]}^{-1}c{\tilde{f}}_{\mathrm{exc}}. \end{array}$$

The displacement and force relations, without the vibration isolators and with the machine source directly connected to the foundation at the former vibration isolator connection points, are $u_{\mathrm{out}}^{\mathrm{no}}=-u_{\mathrm{in}}^{\mathrm{no}}$ and $f_{\mathrm{out}}^{\mathrm{no}}=f_{\mathrm{in}}^{\mathrm{no}}$, where superscript no stands for no vibration isolators. The resulting displacement and force vectors read

(16) $${\tilde{u}}_{\mathrm{out}}^{\mathrm{no}}=-A{\left(BH^{-1}A-\omega^{2}M\right)}^{-1}c{\tilde{f}}_{\mathrm{exc}}$$

and

(17) $${\tilde{f}}_{\mathrm{out}}^{\mathrm{no}}=H^{-1}A{\left(BH^{-1}A-\omega^{2}M\right)}^{-1}c{\tilde{f}}_{\mathrm{exc}},$$

respectively.

The total time averaged energy flow through the vibration isolator bushings into the foundation, while taking into account the directions of the corresponding forces and displacements in Figure 2, is

(18) $$\langle E_{f}\rangle =-\lim_{T\to \infty }\frac{1}{T}\int_{-T/2}^{+T/2}f_{\mathrm{out}}^{T}\left(\tau \right)\frac{d}{d\;\tau }\left[u_{\mathrm{out}}\left(\tau \right)\right]d\;\tau ,$$

which is the same as the cross correlation function

(19) $$S\left(-f_{\mathrm{out}},\frac{d}{d\;t}\left[u_{\mathrm{out}}\right]\right)=-\lim_{T\to \infty }\frac{1}{T}\int_{-T/2}^{+T/2}f_{\mathrm{out}}^{T}\left(\tau \right)\frac{d}{d\;\tau }\left[u_{\mathrm{out}}\left(\tau +t\right)\right]d\;\tau ,$$

evaluated at t=0. However, that is also the same as the inverse temporal Fourier transform of the cross correlation spectral density $\tilde{S}\left(-{\tilde{f}}_{\mathrm{out}},i\omega {\tilde{u}}_{\mathrm{out}}\right)$, evaluated at t=0, [133]

(20) $$\langle E_{f}\rangle =\frac{1}{2\pi }\int_{-\infty }^{\infty }\tilde{S}\left(-{\tilde{f}}_{\mathrm{out}},i\omega {\tilde{u}}_{\mathrm{out}}\right)\exp\left(i\omega t\right){d\;\omega |}_{t=0}=-\int_{-\infty }^{\infty }i2\pi f\tilde{S}\left({\tilde{f}}_{\mathrm{out}},{\tilde{u}}_{\mathrm{out}}\right)d\;f,$$

where the cross correlation spectral density between the force and displacement vector is $\tilde{S}\left({\tilde{f}}_{\mathrm{out}},{\tilde{u}}_{\mathrm{out}}\right)$. Consequently, the total energy flow spectral density [W/Hz], through the vibration isolator bushings into the foundation, is possible to evaluate by the real part *ℜ* of the cross correlation spectral density $ℜ[-i4\pi f\tilde{S}\left({\tilde{f}}_{\mathrm{out}},{\tilde{u}}_{\mathrm{out}}\right)]$, while faulting the energy flow spectral densities at the negative frequencies to the corresponding positive frequencies, using the temporal Fourier transform property $+i\left[-f\right]\tilde{S}\left(-f\right){{|}_{f\geq 0}=-i\left[+f\right]{\tilde{S}}^{*}\left(+f\right)|}_{f\geq 0}$, resulting in $+i\left[-f\right]\tilde{S}{\left(-f\right)}_{\left[\mathrm{negative}\;\mathrm{frequency}:-f\right]}+i\left[+f\right]\tilde{S}{\left(+f\right)}_{\left[\mathrm{positive}\;\mathrm{frequency}:+f\right]}=ℜ[i2f\tilde{S}$${\left(+f\right)]}_{\left[\mathrm{positive}\;\mathrm{frequency}:+f\right]}$, where the complex conjugate operator ${\left(\cdot \right)}^{*}=\mathrm{conj}\left(\cdot \right)$. That is, the total time averaged energy flow trough the vibration isolator bushings into the foundation is

(21) $$\langle E_{f}\rangle =\int_{0}^{\infty }\frac{d\langle E_{f}\rangle }{d\;f}d\;f=-\int_{0}^{\infty }ℜ\left[i4\pi f\tilde{S}\left({\tilde{f}}_{\mathrm{out}},{\tilde{u}}_{\mathrm{out}}\right)\right]d\;f,$$

Ref. [133] while allowing only the positive frequencies. The resulting total energy flow spectral density, through the vibration isolator bushings into the foundation, reads

(22) $$\begin{array}{cc} \frac{d\langle E_{f}\rangle }{d\;f}=ℜ\{ & i4\pi f|K_{\mathrm{out}\;\mathrm{in}}{|}^{2}c^{†}{\left[K_{\mathrm{in}\;\mathrm{in}}BA-K_{\mathrm{out}\;\mathrm{in}}^{2}B{\left(1+K_{\mathrm{out}\;\mathrm{out}}H\right)}^{-1}HA-4\pi^{2}f^{2}M\right]}^{-†}\\ \; & \times A^{†}{\left(1+K_{\mathrm{out}\;\mathrm{out}}H\right)}^{-†}{\left(1+K_{\mathrm{out}\;\mathrm{out}}H\right)}^{-1}HA\\ \; & \times {\left[K_{\mathrm{in}\;\mathrm{in}}BA-K_{\mathrm{out}\;\mathrm{in}}^{2}B{\left(1+K_{\mathrm{out}\;\mathrm{out}}H\right)}^{-1}HA-4\pi^{2}f^{2}M\right]}^{-1}c\}\tilde{S}\left({\tilde{f}}_{\mathrm{exc}},{\tilde{f}}_{\mathrm{exc}}\right), \end{array}$$

by using Equations (12) and (13), where the Hermitian conjugate operator ${\left(\cdot \right)}^{†}=\mathrm{conj}{\left(\cdot \right)}^{T}$, ${\left(\cdot \right)}^{-†}=\mathrm{conj}{\left[{\left(\cdot \right)}^{-1}\right]}^{T}$ and the auto correlation spectral density of the excitation force $\tilde{S}\left({\tilde{f}}_{\mathrm{exc}},{\tilde{f}}_{\mathrm{exc}}\right)\in R^{+}$. Likewise, the resulting total energy flow spectral density, from the machine source into the vibration isolator, bushings reads

(23) $$\begin{array}{cc} \frac{d\langle E_{\mathrm{in}}\rangle }{d\;f}=ℜ\{ & i4\pi fc^{†}{\left[K_{\mathrm{in}\;\mathrm{in}}BA-K_{\mathrm{out}\;\mathrm{in}}^{2}B{\left(1+K_{\mathrm{out}\;\mathrm{out}}H\right)}^{-1}HA-4\pi^{2}f^{2}M\right]}^{-†}\\ \; & \times {\left[K_{\mathrm{in}\;\mathrm{in}}A-K_{\mathrm{out}\;\mathrm{in}}^{2}{\left(1+K_{\mathrm{out}\;\mathrm{out}}H\right)}^{-1}HA\right]}^{†}\\ \; & \times A{\left[K_{\mathrm{in}\;\mathrm{in}}BA-K_{\mathrm{out}\;\mathrm{in}}^{2}B{\left(1+K_{\mathrm{out}\;\mathrm{out}}H\right)}^{-1}HA-4\pi^{2}f^{2}M\right]}^{-1}c\}\tilde{S}\left({\tilde{f}}_{\mathrm{exc}},{\tilde{f}}_{\mathrm{exc}}\right), \end{array}$$

by using the Equations (14) and (15). Finally, the resulting total energy flow spectral density, into the foundation without the vibration isolators, reads  

(24) $$\frac{d\langle E_{f}^{\mathrm{no}}\rangle }{d\;f}=ℜ\left[i4\pi fc^{†}{\left(BH^{-1}A-4\pi^{2}f^{2}M\right)}^{-†}A^{†}H^{-†}A{\left(BH^{-1}A-4\pi^{2}f^{2}M\right)}^{-1}c\right]\tilde{S}\left({\tilde{f}}_{\mathrm{exc}},{\tilde{f}}_{\mathrm{exc}}\right),$$

by using the Equations (16) and (17).

At last, the total energy flow transmissibility spectral density into the foundation

(25) $$\mathrm{EFT}\overset{def}{=}\frac{\frac{d\langle E_{f}\rangle }{d\;f}}{\frac{d\langle E_{f}^{\mathrm{no}}\rangle }{d\;f}}$$

and the corresponding total energy in-flow transmissibility spectral density into the vibration isolator bushings    

(26) $${\mathrm{EFT}}_{\mathrm{in}}\overset{def}{=}\frac{\frac{d\langle E_{\mathrm{in}}\rangle }{d\;f}}{\frac{d\langle E_{f}^{\mathrm{no}}\rangle }{d\;f}},$$

where the nominators and denominators are explicitly given by Equations (22)–(24).

## 3. Results and Discussion

### 3.1. Source, Vibration Isolator Bushing, Foundation and Material Parameters

The dimensions, the mechanical and material parameter quantities and values for the mechanical source, vibration isolator bushings and for the foundation are given in Table 1. The mechanical source is made of aluminum while the foundation is made of steel. The chemical Rouse stress intensity factor C is chosen to be the average value of the materials studied in Kari [33] and with the experiments from Refs. [12,29]. The hydrogel density ρ is close to that of the water and the static modulus $\mu_{\mathrm{st}}$ is possible to modify by selecting a suitable chemical cross-link density [24]. Moreover, the relaxation intensity Δ is possible to modify by selecting a suitable physical-to-chemical cross-link density at the maximum physical cross-link activity [24]. Finally, the frequency for the maximum physical loss shear modulus $f_{a|d}$ is possible to modify by selecting a suitable metal ion to rearrange the adhesion–deadhesion activities of the physical cross-links that in turn result from the alterations in their kinetics and thermodynamics [24]. In passing, borate esterification is the physical cross-link reaction process for the polyvinyl alcohol hydrogels and is thereby somewhat restricting the alteration possibility of the physical cross-link activity [24]. Nonetheless, the energy flow modeling results in this paper are pertinent for a wider class of tough, doubly cross-linked, single network hydrogels with chemical and physical cross-links provided that the hydrogel shear modulus model (5) is applicable.

### 3.2. Hydrogel Shear Modulus

**Figure 3:**
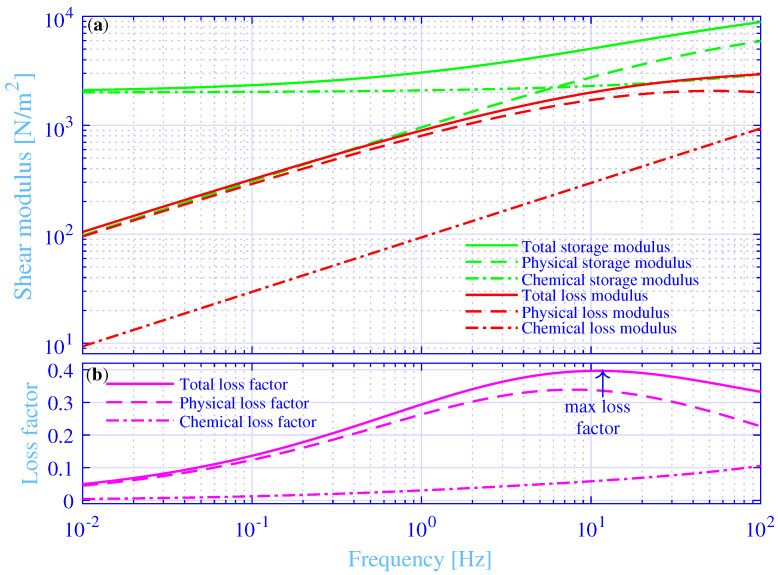
(**a**) The total, the physical and the chemical storage and loss modulus versus the frequency. (**b**) The total, the physical and the chemical loss factor versus the frequency.

The shear modulus and loss factor versus the frequency, in the range 0.01 to 100 Hz, are shown in Figure 3 for the studied hydrogel, while applying $f_{a|d}=52.7\;$Hz. The total storage modulus ℜμ, physical storage modulus $ℜ\mu_{\mathrm{phys}}$ and the chemical storage modulus $ℜ\mu_{\mathrm{chem}}$, are shown in green solid, green dashed and green dash–dotted lines, respectively. Likewise, the total loss modulus ℑμ, physical loss modulus $ℑ\mu_{\mathrm{phys}}$ and the chemical loss modulus $ℑ\mu_{\mathrm{chem}}$, where *ℑ* denotes the imaginary part, are shown in red solid, red dashed and red dash–dotted lines, respectively. Finally, the total loss factor ℑμ/ℜμ, physical loss factor $ℑ\mu_{\mathrm{phys}}/ℜ\mu$ and the chemical loss factor $ℑ\mu_{\mathrm{chem}}/ℜ\mu$, are shown in magenta solid, magenta dashed and magenta dash–dotted lines, respectively. Clearly, the total storage modulus displays an increase with increasing frequency throughout the studied frequency range. It displays a value close to the static modulus $\mu_{\mathrm{st}}$ in the low-frequency end while being close to $\left[1+\Delta \right]\mu_{\mathrm{st}}$ in the high-frequency end. The physical storage modulus displays an increase with increasing frequency throughout the whole considered frequency range. It displays an associated Rouse mode behavior with a half order frequency dependence [39] in the low-frequency range, up to about 1 Hz. The chemical storage modulus displays an almost constant value close to the static modulus $\mu_{\mathrm{st}}$ in the low-frequency range, up to about 1 Hz, then it increases slightly with increasing frequency.

The total and physical loss modulus display an associated Rouse mode behavior in the low-frequency range, up to about 1 Hz. Then they flatten out for even higher frequencies. However, the total loss modulus continues to increase while the corresponding physical loss modulus displays a maximum of $\mu_{\mathrm{st}}\Delta /2\left(1+\sqrt{2}\right)=2070\;$N/m${}^{2}$ at $f_{a|d}=52.7\;$Hz. The physical loss modulus decreases with increasing frequencies above that frequency point. The adhesion–deadhesion activities of the physical cross-links are at their maximum at $f_{a|d}$, that results in a maximum physical loss modulus. Physically, the time frame for profoundly lower frequencies $f\ll f_{a|d}$ is long—thus, allowing for early debonding of most of the physical cross-links and results in a lower physical loss and storage modulus. Conversely, the time frame for profoundly higher frequencies $f\gg f_{a|d}$ is short, thus not allowing for debonding of most of the physical cross-links and results also in a lower physical loss modulus. Furthermore, it results in a higher physical storage modulus, since a majority of the physical cross-links are bonded throughout the whole time frame. More details of the physical explanations are given in such as Refs. [12,14,24,33], for the interested reader. The chemical loss modulus is significantly smaller than the corresponding total and physical loss modula. It displays an associated Rouse mode behavior throughout the whole studied frequency range. The main contribution of the chemical cross-links is to the storage modulus, in particular at the low-frequency range, where it is the dominating cross-link contribution to the total shear modulus. As a result, the total and physical loss modulus are close to each other up to about 10 Hz, where they start to deviate with increasing frequency.

Likewise, the total and physical loss factors are close to each other up to about 1 Hz, where they start to deviate with increasing frequency. They display an increase with increasing frequency in the low-frequency range while showing the opposite in the high-frequency range. That is, they display a decrease with increasing frequency. As a result, they show a maximum loss factor in-between those frequency ranges. The total loss factor shows a maximum of about 0.396 at $f_{\max[\eta ]}=11.0\;$Hz, while the corresponding maximum physical loss factor is about 0.339 at $f_{\max[\eta_{\mathrm{phys}}]}=7.81\;$Hz. In passing, the maximum physical loss factor is $\Delta /[{\left(\sqrt{2}+\sqrt{1+\Delta }\right)}^{2}-1]=0.359$ at $f_{\max[\eta_{\mathrm{phys}}\left(C=0\right)]}=f_{a|d}/\left(1+\Delta \right)=8.78\;$Hz, while redefining the physical loss factor as $ℑ\mu_{\mathrm{phys}}/ℜ\mu \left(C=0\right)$. The chemical loss factor displays a slight increase with increasing frequency. It is substantially smaller than the corresponding total and physical loss factors, up to about 10 Hz, where they begin to approach each other with increasing frequency.

### 3.3. Dynamic Stiffness

**Figure 4:**
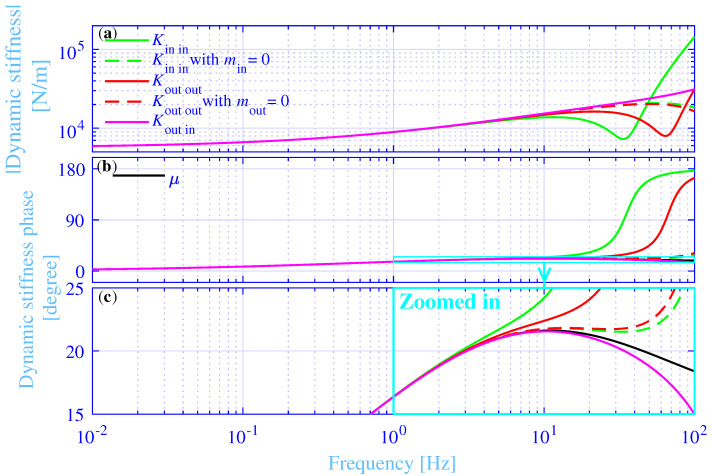
(**a**) The absolute value of the axial inner driving point, outer driving point and transfer dynamic stiffness versus the frequency, with $f_{a|d}=52.7\;$Hz. (**b**) The corresponding phase of the axial inner driving point, outer driving point and transfer dynamic stiffness versus the frequency. (**c**) The zoomed in version of the phase of the axial dynamic stiffness in (**b**) versus the frequency.

The absolute value of the axial dynamic stiffness |K| and its phase ∠K, fulfilling K=|K|exp(i∠K), versus the frequency, in the range 0.01 to 100 Hz, are shown in Figure 4 for the studied hydrogel vibration isolator bushings, while applying $f_{a|d}=52.7\;$Hz. The axial inner dynamic driving point stiffness $K_{\mathrm{in}\;\mathrm{in}}$, is shown in green solid lines, the axial dynamic outer driving point stiffness $K_{\mathrm{out}\;\mathrm{out}}$ is shown in red solid lines and the axial dynamic transfer stiffness $K_{\mathrm{out}\;\mathrm{in}}$, is shown in magenta solid lines. The corresponding axial inner and outer dynamic driving point stiffness, while assuming weightless inner and outer metal sleeves $m_{\mathrm{in}}=m_{\mathrm{out}}=0$, are shown in green and red dashed lines, respectively. Clearly, the inner and outer driving point stiffness and the transfer stiffness almost overlap in the low-frequency range, up to just over 1 Hz. That overlap holds for both their absolute values and their phases, respectively. The absolute values increase with increasing frequency up to about 10 Hz. Then the inner and outer driving point stiffness drop. First the inner driving point stiffness is dropping into a trough at about 34 Hz, followed by the outer driving point stiffness that is dropping into a trough at about 64 Hz. Then they rise again with increasing frequency. The trough location at a lower frequency for the inner driving point stiffness compared to the trough location for the outer driving point stiffness is due to a larger inner metal sleeve mass $m_{\mathrm{in}}>m_{\mathrm{out}}$. The corresponding inner and outer driving point stiffnesses, with weightless inner and outer metal sleeves, drop at higher frequencies without showing any trough within the considered frequency range. The transfer stiffness displays an increase with increasing frequency throughout the whole considered frequency range, without any drop, nor a trough. The drops and troughs of the inner and outer driving point stiffness are mainly due to resonances of the spring–mass systems, where the spring is the hydrogel vibration isolator bushing with weightless inner and outer metal sleeves, and the mass is the mass of the inner and outer metal sleeves, respectively. That conclusion is verified by the approximate +180 degree jump of the driving point stiffness phases around the trough frequencies in Figure 4b. On the contrary, the drops of the inner and outer driving point stiffness, with weightless inner and outer metal sleeves, at the higher frequencies are instead due to the wave effects within the hydrogel vibration isolator bushing, eventually showing internal resonances at even higher frequencies, outside the considered frequency range. This is verified by the sharp rise of their stiffness phases at the high-frequency end of the zoomed area in Figure 4c. The conclusion is furthermore verified by the deviating stiffness phases starting from just over 1 Hz, where in addition the shear modulus phase, ∠μ is shown in black solid line, fulfilling μ=|μ|exp(i∠μ). Any stiffness phase deviation from the shear modulus phase indicates either internal wave or mass–spring effects, eventually leading to internal or mass–spring resonances or anti-resonances. Finally, the inner and outer driving point phases are thermodynamically required to fulfill 0≤∠K≤π. This requirement is clearly fulfilled within the considered frequency range.

### 3.4. Energy Flow Transmissibility

**Figure 5:**
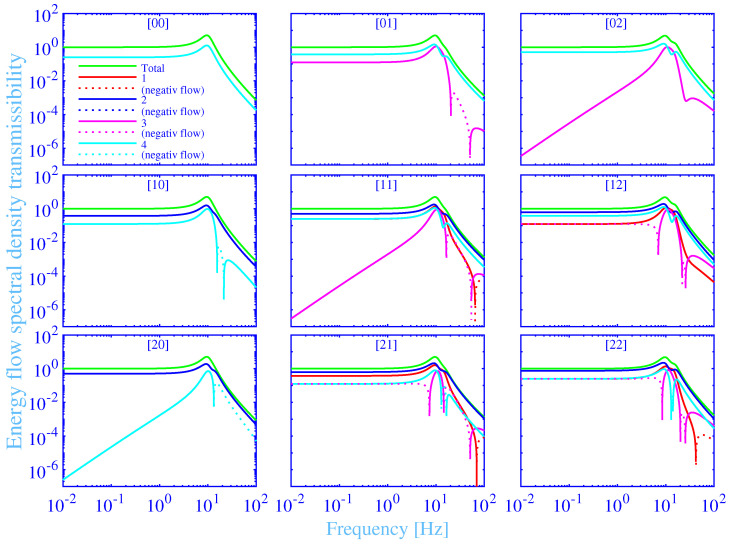
The total and individual energy flow transmissibility spectral densities into the foundation versus the frequency, at the force excitation points [n m], with n,m=0,1,2 .

The energy flow transmissibility spectral density into the foundation versus the frequency, in the range 0.01 to 100 Hz, is shown in Figure 5 for the studied hydrogel vibration isolation system in Figure 1. The results for all the nine combinations of the force excitation points [n m], with n,m=0,1,2 , at the upper surface of the machine source, according to Figure 1, are shown as subplots. The total energy flow transmissibility spectral density into the foundation EFT, is shown in green solid lines. The individual energy flow transmissibility spectral densities into the foundation ${\mathrm{EFT}}_{ⓙ}$, j=1,2,3,4 , through the hydrogel vibration isolator bushing **➀**, **➁**, **➂** and **➃** (see Figure 1), are shown in red, blue, magenta and cyan solid lines, respectively. The relation $\mathrm{EFT}={\mathrm{EFT}}_{➀}+{\mathrm{EFT}}_{➁}+{\mathrm{EFT}}_{➂}+{\mathrm{EFT}}_{➃}$ holds, since all the energy flow transmissibility spectral densities are calculated in relation to the total energy flow spectral density into the foundation, without the vibration isolators. Negative energy flow transmissibility spectral densities, at reversed energy flows, directed from the foundation into the vibration isolators, are shown in dotted lines. The excitation force may excite up to three rigid body resonances of the studied hydrogel vibration isolation system, with their motions appropriately described by the generalized displacement vector $\tilde{u}={\left(\tilde{U},{\tilde{W}}_{x},{\tilde{W}}_{y}\right)}^{T}$. The main idea in this paper is to introduce the tough, single network hydrogel vibration isolator bushings with chemical and physical cross-links to reduce the energy flow transmissibility into the foundation—in particular, to reduce this transmissibility at the rigid body resonances of the vibration isolation system. The latter is an essential challenge and disadvantage for the more traditional vibration isolation systems, while using such as natural rubber vibration isolators. To this end, the maximum of the energy flow transmissibility spectral density ${\mathrm{EFT}}_{\max}$, from Equation (25), should be minimized. This is performed by finding an optimal frequency for the maximum physical loss shear modulus $f_{a|d}^{\mathrm{opt}}$, while using the constrained non-linear multi-variable programming solver fmincon from Matlab${}^{®}$ [134]. The resulting $f_{a|d}^{\mathrm{opt}}$, for each force excitation point [n m], are shown in Table 2, with the corresponding minimum value of the maximum energy flow transmissibility spectral density $\min[{\mathrm{EFT}}_{\max}]$ and the frequency for the maximum energy flow transmissibility spectral density $f_{\max}$. Moreover, the maximum value of the energy in-flow transmissibility spectral density ${\mathrm{EFT}}_{\mathrm{in}\;\max}$ (see Section 3.5) and the corresponding frequency for the maximum energy in-flow transmissibility spectral density $f_{\mathrm{in}\;\max}$, are shown in Table 2. Furthermore, the resulting frequency for the maximum total loss factor $f_{\max[\eta ]}$, for each force excitation point [n m], are shown in Table 2, with the corresponding frequency for the maximum physical loss factor $f_{\max[\eta_{\mathrm{phys}}]}$ and, finally, the frequency for the maximum (redefined) physical loss factor, while using C=0, in parenthesis.

Clearly, there are at most three total energy flow transmissibility spectral density peaks in the subplots of Figure 5—each corresponding to a rigid body resonance of the vibration isolation system. The first rigid body resonance at about 9.7 Hz, is linked to the rectilinear motion $\tilde{U}$ parallel to the *z*-axis, the second rigid body resonance at about 13 Hz, is linked to the rotating motion ${\tilde{W}}_{y}$ around the *y*-axis and the third rigid body resonance at about 15 Hz, is linked to the rotating motion ${\tilde{W}}_{x}$ around the *x*-axis. The force excitation at the point [0 0], results only in a single rigid body resonance excited—namely that linked to the rectilinear motion $\tilde{U}$ parallel to the *z*-axis and is due to the double symmetry with respect to both the *x*- and *y*-axis. The number of rigid body resonances excited is increased to two, at the force excitations at the points [1 0] and [2 0], by including also the rigid body resonance linked to the rotating motion ${\tilde{W}}_{y}$ around the *y*-axis, and is due to the symmetry reduction to include only the symmetry with respect the *x*-axis. Likewise, the number of rigid body resonances excited is increased to two at the force excitations at the points [0 1] and [0 2], by including also the rigid body resonance linked to the rotating motion ${\tilde{W}}_{x}$ around the *x*-axis, and is due to the symmetry reduction to include only the symmetry with respect the *y*-axis. Finally, the number of rigid body resonances excited is increased to three, at the force excitations at the points [1 1], [2 1], [1 2] and [2 2], by including also the rigid body resonances linked to the rotating motions ${\tilde{W}}_{x}$ and ${\tilde{W}}_{y}$ around the *x*- and *y*-axis, respectively, and is due to the symmetry reduction into no symmetry. The dominating rigid body resonance, from the maximum total energy flow transmissibility spectral density point of view, including all the force excitation point considered in this study, is the rigid body resonance linked to the rectilinear motion $\tilde{U}$ parallel to the *z*-axis, and is displaying the highest peaks in all the subplots of Figure 5. Furthermore, the increased number of rigid body resonances excited result in an increased distribution of the total energy flow into more degrees-of-freedom that, in turn, result in a decreased maximum total energy flow transmissibility spectral density peaks for the dominating degree-of-freedom. That conclusion is clearly obvious in the subplots of Figure 5 and, in particular, in the Table 2 that shows that the maximum total energy flow transmissibility spectral density peak reduces from 5.09 for the excitation force at [0 0], to 5.06 for the excitation force at [2 0], to 4.83 for the excitation force at [0 2] and to 4.82 for the excitation force at [2 2]. Moreover, the rigid body resonances are strongly overlapping due to close rigid body resonance frequencies and due to the high damping of the tough, doubly cross-linked, single network hydrogel vibration isolator bushings with chemical and physical cross-links. The strong overlap results in a shift of the frequency for the maximum energy flow transmissibility spectral density $f_{\max}$ into slightly higher frequencies. This frequency shift increases with increasing excitation of the rotating rigid body resonances, as is clearly noticeable in Table 2, where $f_{\max}=9.49\;$Hz for the excitation force at [0 0], increases to 9.50 Hz for the excitation force at [2 0], increases to 9.55 Hz for the excitation force at [0 2] and increases to 9.56 Hz for the excitation force at [2 2]. The latter excitation force excites both the rotating rigid body resonances, in addition to the rectilinear rigid body resonance of the vibration isolation system. However, the strong overlap makes it difficult to visually dissolve all the three peaks simultaneously.

The total energy flow transmissibility spectral density into the foundation EFT, for the excitation force at [0 0] in Figure 5, displays almost a constant value close to 1 in the low-frequency range, up to about 1 Hz. Then it rises with increasing frequency and shows a maximum of ${\mathrm{EFT}}_{\max}=5.09$ at $f_{\max}=9.49\;$Hz. Then it drops with increasing frequency, displays EFT=1 at about f=14.3 Hz and continues to drop with increasing frequency, displaying $\mathrm{EFT}=7.22\times \;10^{-4}$ at the high-frequency end, f=100 Hz. The conclusion is that the studied vibration isolation system functions as intended for the frequencies above 14.3 Hz and shows, in addition, a fast vibration isolation improvement with increasing frequency above that frequency. However, and as already mentioned, the total energy flow transmissibility spectral density into the foundation at a rigid body resonance of the vibration isolation system is important since, in practice, it is generally unavoidable to excite a rigid body resonance in a real vibration isolation system—that is, to avoid excitation force frequencies below 14.3 Hz. This can happen at the starting up, turning off and at changing the revolutions per minute of machines, among other circumstances. The corresponding individual energy flow transmissibility spectral densities into the foundation ${\mathrm{EFT}}_{ⓙ}$, j=1,2,3,4 , are exactly overlapping while showing ${\mathrm{EFT}}_{ⓙ}=\mathrm{EFT}/4$. This is not surprising as the vibration isolation system is displaying a double symmetry with respect to both the *x*- and *y*-axis, for the excitation force at [0 0].

The individual energy flow transmissibility spectral densities into the foundation, for the excitation force at [1 0] and [2 0] in Figure 5, are grouped into ${\mathrm{EFT}}_{➀}={\mathrm{EFT}}_{➁}$ and ${\mathrm{EFT}}_{➂}={\mathrm{EFT}}_{➃}$, due to the symmetry with respect to the *x*-axis. The dominating individual energy flow transmissibility spectral densities are ${\mathrm{EFT}}_{➀}$ and ${\mathrm{EFT}}_{➁}$, displaying an almost a constant value of about 0.38 for the excitation force at [1 0] and about 0.50 for the excitation force at [2 0], in the low-frequency range, up to about 1 Hz. The corresponding individual energy flow transmissibility spectral densities ${\mathrm{EFT}}_{➂}$ and ${\mathrm{EFT}}_{➃}$ display an almost a constant value of about 0.13 in the low-frequency range, up to about 1 Hz, for the excitation force at [1 0], while they display a very small value of $2.5\times 10^{-7}$ at the low-frequency end, f=0.01 Hz, for the excitation force at [2 0]. The latter individual energy flow transmissibility spectral densities increase then fast with increasing frequency and shows $1.67\times 10^{-3}$ at f=1 Hz, for the excitation force at [2 0]. Interestingly, the individual energy flow transmissibility spectral densities ${\mathrm{EFT}}_{➂}$ and ${\mathrm{EFT}}_{➃}$ display also a reversed energy flow approximately between the frequencies 15.7 and 21.4 Hz, for the excitation force at [1 0], and from 13.4 Hz to the high-frequency end, f=100 Hz, for the excitation force at [2 0].

Likewise, the individual energy flow transmissibility spectral densities into the foundation, for the excitation force at [0 1] and [0 2] in Figure 5, are grouped into ${\mathrm{EFT}}_{➀}={\mathrm{EFT}}_{➂}$ and ${\mathrm{EFT}}_{➁}={\mathrm{EFT}}_{➃}$, due to the symmetry with respect to the *y*-axis. The dominating individual energy flow transmissibility spectral densities are ${\mathrm{EFT}}_{➁}$ and ${\mathrm{EFT}}_{➃}$, displaying an almost a constant value of about 0.38 for the excitation force at [0 1] and about 0.50 for the excitation force at [0 2], in the low-frequency range, up to about 1 Hz. The corresponding individual energy flow transmissibility spectral densities ${\mathrm{EFT}}_{➀}$ and ${\mathrm{EFT}}_{➂}$ display an almost a constant value of about 0.12 in the low-frequency range, up to about 1 Hz, for the excitation force at [0 1], while they display a very small value of $3.45\times 10^{-7}$ at the low-frequency end, f=0.01 Hz, for the excitation force at [0 2]. The latter individual energy flow transmissibility spectral densities increase fast with increasing frequency and show $2.25\times 10^{-3}$ at f=1 Hz, for the excitation force at [0 2]. Furthermore, the individual energy flow transmissibility spectral densities ${\mathrm{EFT}}_{➀}$ and ${\mathrm{EFT}}_{➂}$ display also a reversed energy flow approximately between the frequencies 20.4 and 50.5 Hz, for the excitation force at [0 1].

Furthermore, the individual energy flow transmissibility spectral densities into the foundation, for the excitation force at [2 2] in Figure 5, are fully split into the separate individual contributions ${\mathrm{EFT}}_{➀}$, ${\mathrm{EFT}}_{➁}$, ${\mathrm{EFT}}_{➂}$ and ${\mathrm{EFT}}_{➃}$, due to the lacking symmetries with respect to the *x*- and *y*-axis. The dominating individual energy flow transmissibility spectral density is ${\mathrm{EFT}}_{➁}$, displaying an almost a constant value of about 0.75, in the low-frequency range, up to about 1 Hz. The corresponding individual energy flow transmissibility spectral densities ${\mathrm{EFT}}_{➀}$ and ${\mathrm{EFT}}_{➃}$ display an almost a constant value of about 0.25 in the low-frequency range, up to about 1 Hz, while the corresponding individual energy flow transmissibility spectral density ${\mathrm{EFT}}_{➂}$ is displaying a reversed energy flow with an almost a constant negative value of about −0.25, in the low-frequency range, up to about 1 Hz. In fact, the reversed, negative individual energy flow transmissibility spectral density ${\mathrm{EFT}}_{➂}$ is extended to the frequency of about 8.42 Hz, where it change the sign to a positive energy flow. Furthermore, the same individual energy flow transmissibility spectral density ${\mathrm{EFT}}_{➂}$ displays a second frequency range of a reversed, negative individual energy flow transmissibility spectral density, approximately between the frequencies 20.1 and 25.3 Hz. At last, the individual energy flow transmissibility spectral density ${\mathrm{EFT}}_{➃}$ displays a reversed, negative individual energy flow transmissibility spectral density approximately between the frequencies 13.0 and 14.0 Hz, while the individual energy flow transmissibility spectral density ${\mathrm{EFT}}_{➀}$ displays accordingly a reversed, negative individual energy flow transmissibility spectral density approximately from 42.1 Hz to the high-frequency end, f=100 Hz.

Finally, the total energy flow transmissibility spectral density into the foundation is thermodynamically required to be non-negative. This requirement is clearly fulfilled, within the considered frequency range, for all the force excitation points [n m], n,m=0,1,2 , in Figure 5.

### 3.5. Energy In-Flow Transmissibility

**Figure 6:**
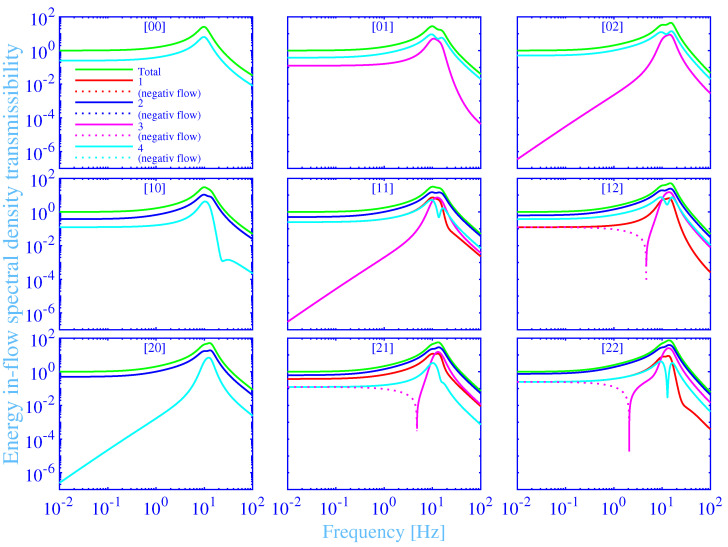
The total and individual energy in-flow transmissibility spectral densities into the vibration isolator bushings versus the frequency, at the force excitation points [n m], with n,m=0,1,2 .

The energy in-flow transmissibility spectral density into the vibration isolator bushings versus the frequency, in the range 0.01 to 100 Hz, is shown in Figure 6. The results for all the nine combinations of the force excitation points [n m], with n,m=0,1,2 , are shown as subplots. The total energy in-flow transmissibility spectral density into the vibration isolator bushings ${\mathrm{EFT}}_{\mathrm{in}}$, is shown in green solid lines. The individual energy in-flow transmissibility spectral densities into a single vibration isolator bushing ${\mathrm{EFT}}_{\mathrm{in}\;ⓙ}$, j=1,2,3,4 , for the hydrogel vibration isolator bushing **➀**, **➁**, **➂** and **➃** are shown in red, blue, magenta and cyan solid lines, respectively. The relation ${\mathrm{EFT}}_{\mathrm{in}}={\mathrm{EFT}}_{\mathrm{in}\;➀}+{\mathrm{EFT}}_{\mathrm{in}\;➁}+{\mathrm{EFT}}_{\mathrm{in}\;➂}+{\mathrm{EFT}}_{\mathrm{in}\;➃}$ holds since all the energy in-flow transmissibility spectral densities are calculated in relation to the total energy flow spectral density into the foundation, without the vibration isolators. Negative energy in-flow transmissibility spectral densities, at reversed energy flows, directed from the vibration isolator bushings into the mechanical source, are shown in dotted lines.

The total and individual energy in-flow transmissibility spectral densities into the vibration isolator bushings in Figure 6 show similar frequency dependence as the corresponding energy flow transmissibility spectral densities into the foundation. However, the maximum total energy in-flow transmissibility spectral densities are showing larger peak values. For example, the maximum total energy in-flow transmissibility spectral density into the vibration isolator bushings, for the excitation force at [0 0], is ${\mathrm{EFT}}_{\mathrm{in}\;\max}=25.5$ as compared to the corresponding ${\mathrm{EFT}}_{\max}=5.09$. That is, part of the energy flow into the vibration isolator bushings is absorbed in the bushing and transformed into heat, while the remaining part of the energy flow is conveyed further into the foundation. In other words, the vibration isolator bushings also act as vibration dampers, transforming the mechanical energy into heat. In fact, ${\mathrm{EFT}}_{\mathrm{in}}\geq \mathrm{EFT}$ holds since the heat produced must be non-negative from the thermodynamical point of view. The equality relation ${\mathrm{EFT}}_{\mathrm{in}}=\mathrm{EFT}$ is only possible for purely elastic vibration isolator materials, without any losses. The corresponding relations hold also for the individual energy flows ${\mathrm{EFT}}_{\mathrm{in}\;ⓙ}\geq {\mathrm{EFT}}_{ⓙ}$, j=1,2,3,4 . Moreover, the frequencies for the maximum total energy in-flow transmissibility spectral densities are shifted to slightly higher frequencies in Figure 6, as compared to the corresponding frequencies for the maximum total energy flow transmissibility spectral densities in Figure 5, for all the force excitation points [n m], n,m=0,1,2 . For example, the frequency for the maximum total energy in-flow transmissibility spectral density, for the excitation force at [0 0], is shifted from $f_{\max}=9.49\;$Hz to $f_{\mathrm{in}\;\max}=9.75\;$Hz. Furthermore, the dominating rigid body resonance, from the maximum total energy in-flow transmissibility spectral density point of view, is the rigid body resonance linked to the rectilinear motion parallel to the *z*-axis for the excitation force at [0 0], [1 0], [0 1] and [1 1]. The result corresponds to the results for the dominating total energy flow transmissibility spectral density. However, the results for the excitation force at [0 2] and [1 2] are mainly linked to the rotating motion around the *x*-axis, while the results for the excitation force at [2 0], [2 1] and [2 2] are mainly linked to the rotating motion around the *y*-axis. The latter excitation force point shows, in addition, a strong overlap with the rigid body resonance linked to the rotating motion around the *x*-axis.

The total energy in-flow transmissibility spectral density into the vibration isolator bushings is thermodynamically required to be non-negative. This requirement is clearly fulfilled within the considered frequency range for all the force excitation points [n m], n,m=0,1,2 , in Figure 6. However, the individual energy in-flow transmissibility spectral densities into the vibration isolator bushings are possible to be negative, like the corresponding individual energy flow transmissibility spectral densities into the foundation. In fact, the individual energy in-flow transmissibility spectral density ${\mathrm{EFT}}_{\mathrm{in}\;➂}$, into the vibration isolator bushing **➂**, displays a reversed energy flow from the low-frequency end, f=0.01 Hz to 4.77, 4.69 and to 2.08 Hz, for the excitation force at [2 1], [1 2] and [2 2], respectively. In passing, the thermodynamically grounded individual energy in-flow transmissibility spectral density relations imply, among other things, that the energy in-flow is not possible to be reversed concurrently with a positive energy flow into the foundation, for each individual vibration isolator bushing. This requirement is clearly fulfilled for all vibration isolator bushings in Figure 5 and Figure 6.

### 3.6. Minimum Analysis

**Figure 7:**
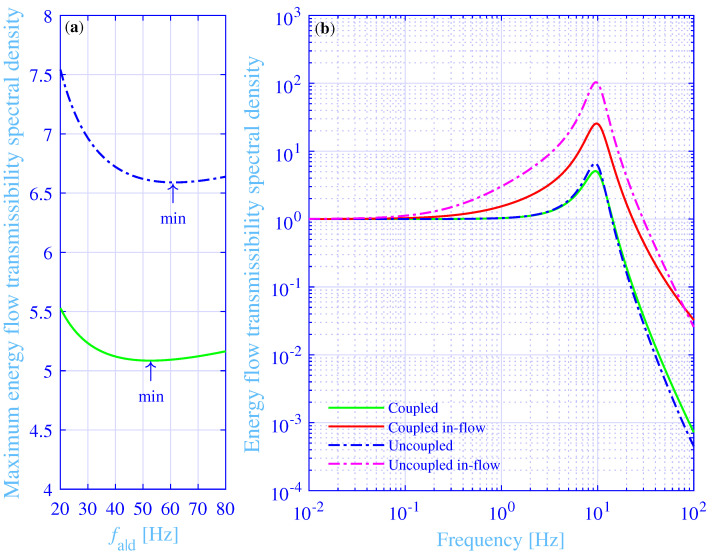
(**a**) The maximum energy flow transmissibility spectral densities versus the frequency for the maximum physical loss modulus, for the coupled and uncoupled hydrogel vibration isolation systems. (**b**) The energy flow and in-flow transmissibility spectral densities versus the frequency, for the coupled and uncoupled hydrogel vibration isolation systems.

The first and second order derivatives of the energy flow transmissibility spectral density EFT, for the excitation force at [0 0], with respect to the frequency for the maximum physical loss shear modulus $f_{a|d}$ at its optimum value $f_{a|d}^{\mathrm{opt}}=52.7\;$Hz, while using the central finite difference scheme of $8^{\mathrm{th}}$-order accuracy from Fornberg [135], with a uniform step-size $\Delta_{a|d}$,

(27) $$\begin{array}{cc} \frac{d[\mathrm{EFT}\left(f_{a|d}^{\mathrm{opt}}\right)]}{d\;f_{a|d}^{\mathrm{opt}}}=\frac{1}{\Delta_{a|d}}[ & \frac{1}{280}\mathrm{EFT}\left(f_{a|d}^{\mathrm{opt}}-4\Delta_{a|d}\right)-\frac{4}{105}\mathrm{EFT}\left(f_{a|d}^{\mathrm{opt}}-3\Delta_{a|d}\right)+\frac{1}{5}\mathrm{EFT}\left(f_{a|d}^{\mathrm{opt}}-2\Delta_{a|d}\right)\\ \; & -\frac{4}{5}\mathrm{EFT}\left(f_{a|d}^{\mathrm{opt}}-\Delta_{a|d}\right)+\frac{4}{5}\mathrm{EFT}\left(f_{a|d}^{\mathrm{opt}}+\Delta_{a|d}\right)-\frac{1}{5}\mathrm{EFT}\left(f_{a|d}^{\mathrm{opt}}+2\Delta_{a|d}\right)\\ \; & +\frac{4}{105}\mathrm{EFT}\left(f_{a|d}^{\mathrm{opt}}+3\Delta_{a|d}\right)-\frac{1}{280}\mathrm{EFT}\left(f_{a|d}^{\mathrm{opt}}+4\Delta_{a|d}\right)]+O\left(\Delta_{a|d}^{8}\right) \end{array}$$

and

(28) $$\begin{array}{cc} \frac{d^{2}\left[\mathrm{EFT}\left(f_{a|d}^{\mathrm{opt}}\right)\right]}{{\left[d\;f_{a|d}^{\mathrm{opt}}\right]}^{2}}=\frac{1}{\Delta_{a|d}^{2}}[ & -\frac{1}{560}\mathrm{EFT}\left(f_{a|d}^{\mathrm{opt}}-4\Delta_{a|d}\right)+\frac{8}{315}\mathrm{EFT}\left(f_{a|d}^{\mathrm{opt}}-3\Delta_{a|d}\right)-\frac{1}{5}\mathrm{EFT}\left(f_{a|d}^{\mathrm{opt}}-2\Delta_{a|d}\right)\\ \; & +\frac{8}{5}\mathrm{EFT}\left(f_{a|d}^{\mathrm{opt}}-\Delta_{a|d}\right)-\frac{205}{72}\mathrm{EFT}\left(f_{a|d}^{\mathrm{opt}}\right)+\frac{8}{5}\mathrm{EFT}\left(f_{a|d}^{\mathrm{opt}}-\Delta_{a|d}\right)\\ \; & -\frac{1}{5}\mathrm{EFT}\left(f_{a|d}^{\mathrm{opt}}+2\Delta_{a|d}\right)+\frac{8}{315}\mathrm{EFT}\left(f_{a|d}^{\mathrm{opt}}+3\Delta_{a|d}\right)\\ \; & -\frac{1}{560}\mathrm{EFT}\left(f_{a|d}^{\mathrm{opt}}+4\Delta_{a|d}\right)]+O\left(\Delta_{a|d}^{8}\right), \end{array}$$

read $|d\left[\mathrm{EFT}\left(f_{a|d}^{\mathrm{opt}}\right)\right]/d\;f_{a|d}^{\mathrm{opt}}|<10^{-5}\;$Hz${}^{-1}\approx 0$ and $d^{2}\left[\mathrm{EFT}\left(f_{a|d}^{\mathrm{opt}}\right)\right]/{\left(d\;f_{a|d}^{\mathrm{opt}}\right)}^{2}=+3.28\times 10^{-4}\;$Hz${}^{-2}$, respectively. That is, the energy flow transmissibility spectral density clearly displays a minimum at $f_{a|d}^{\mathrm{opt}}$. A numerical step-size experimentation for this classical ill-conditioned numerical differentiation try-out reveals that a trade-off between a large subtraction rounding error, due to an overly small step-size, and large first and second derivative estimation method errors, due to an overly large step-size, lies approximately between $10^{-1}\;\mathrm{Hz}\leq \Delta_{a|d}\leq 10^{-4}\;$Hz, while using binary64 (double) precision calculations according to the IEEE Standard 754-2019 revision for the floating-point arithmetic. In fact, the maximum energy flow transmissibility spectral density results in Figure 7a, versus the frequency for the maximum physical loss shear modulus, reveal that the optimum value $f_{a|d}^{\mathrm{opt}}=52.7\;$Hz results actually in a minimum of the maximum energy flow transmissibility spectral density into the foundation.

The frequency for the maximum energy flow transmissibility spectral density $f_{\max}$, for all the force excitation points in Table 2, is located close to the frequency for the maximum loss factor $f_{\max[\eta ]}$, still not exactly at that frequency. For example, the frequency difference $f_{\max[\eta ]}-f_{\max}=1.50\;$Hz, for the force excitation at [0 0]. This difference decreases with increasing number of rigid body resonances excited. For example, the frequency difference $f_{\max[\eta ]}-f_{\max}$ decreases from 1.50 to 1.47, 1.03 and 1.01 Hz, for the force excitation at [0 0] to [2 0], [0 2] and [2 2]. The main reasons for the frequency difference decrease are the decreased frequency for the maximum loss factor and the increased frequency for the maximum energy flow transmissibility spectral density, with the increasing number of rigid body resonances excited.

A straightforward method to further explore the minimum obtained for the maximum energy flow transmissibility spectral density, is to reduce the complexity of the studied vibration isolation system. To this end, the coupling between the vibration isolator connection points to the foundation is disregarded. That is, the off-diagonal elements of the compliance matrix H are set to zero in Equation (9). As a result, the uncoupled total energy flow transmissibility spectral density, for the excitation force at [0 0], reads

(29) $${\mathrm{EFT}}_{\mathrm{uncoupled}}={\left|\frac{K_{\mathrm{out}\;\mathrm{in}}\left(K_{f}-\pi^{2}f^{2}M\right)}{\left(K_{\mathrm{in}\;\mathrm{in}}-\pi^{2}f^{2}M\right)\left(K_{f}+K_{\mathrm{out}\;\mathrm{out}}\right)-K_{\mathrm{out}\;\mathrm{in}}^{2}}\right|}^{2},$$

where the uncoupled dynamic stiffness of the foundation $K_{f}=16i\pi fh^{2}\sqrt{\Upsilon_{f}\rho_{f}/12\left(1-\nu^{2}\right)}$, being identical to the inverse of the diagonal elements of H in Equation (9). The total energy flow and energy in-flow transmissibility spectral densities versus the frequency, in the range 0.01 to 100 Hz, are shown in Figure 7b, for both the fully coupled and uncoupled foundation, while applying $f_{a|d}^{\mathrm{opt}}=52.7\;$Hz and $f_{a|d\;\mathrm{uncoupled}}^{\mathrm{opt}}=61.4\;$Hz, respectively. Furthermore, the maximum total energy flow transmissibility spectral density versus the frequency for the maximum physical loss shear modulus, is also shown in Figure 7a, for the uncoupled foundation. Clearly, the optimal value $f_{a|d\;\mathrm{uncoupled}}^{\mathrm{opt}}=61.4\;$Hz results in a minimum of the maximum total energy flow transmissibility spectral density into the foundation. Moreover, the maximum loss factor $\eta_{\mathrm{uncoupled}}=0.399$ at the frequency $f_{\max[\eta_{\mathrm{uncoupled}}]}=13.0\;$Hz. In addition, the maximum total energy flow transmissibility spectral density ${\mathrm{EFT}}_{\max\;\mathrm{uncoupled}}=6.59$ at the frequency $f_{\max\;\mathrm{uncoupled}}=9.41\;$Hz. Clearly, this frequency is, as for the fully coupled vibration isolation system, located rather close to the frequency for the maximum loss factor. The corresponding frequency difference reads $f_{\max[\eta_{\mathrm{uncoupled}}]}-f_{\max\;\mathrm{uncoupled}}=3.62\;$Hz. Furthermore, the frequency $f=f_{\max\;\mathrm{uncoupled}}^{\mathrm{appr}}$ that satisfies

(30) $$ℜ\left\{K_{\mathrm{in}\;\mathrm{in}}\left(f_{\max\;\mathrm{uncoupled}}^{\mathrm{appr}}\right)-\pi^{2}{\left[f_{\max\;\mathrm{uncoupled}}^{\mathrm{appr}}\right]}^{2}M\right\}=0,$$

namely $f_{\max\;\mathrm{uncoupled}}^{\mathrm{appr}}=9.51\;$Hz, is close to the frequency for the maximum total energy flow transmissibility spectral density into the foundation $f_{\max\;\mathrm{uncoupled}}=9.41\;$Hz. The Equation (30) forces the real part of a factor in a term of the denominator in Equation (29) to vanish. The resulting maximum total energy flow transmissibility spectral density

(31) $${\mathrm{EFT}}_{\max\;\mathrm{uncoupled}}^{\mathrm{appr}}={\left|\frac{K_{\mathrm{out}\;\mathrm{in}}\left(K_{f}-\pi^{2}f^{2}M\right)}{i\;ℑ\left(K_{\mathrm{in}\;\mathrm{in}}\right)\left(K_{f}+K_{\mathrm{out}\;\mathrm{out}}\right)-K_{\mathrm{out}\;\mathrm{in}}^{2}}\right|}^{2}=6.58,$$

at $f=f_{\max\;\mathrm{uncoupled}}^{\mathrm{appr}}$, and is close to the maximum total energy flow transmissibility spectral density into the foundation ${\mathrm{EFT}}_{\max\;\mathrm{uncoupled}}=6.59$ at the frequency $f_{\max\;\mathrm{uncoupled}}=9.41\;$Hz. Moreover, assume the following relations to hold at $f=f_{\max\;\mathrm{uncoupled}}^{\mathrm{appr}}$, with the specific value outcomes in brackets:

1. $|K_{f}\left|\gg \right|K_{\mathrm{out}\;\mathrm{out}}|$                                    [$5.85\times 10^{5}\;$N/m${}^{2}\gg 1.41\times 10^{4}\;$N/m${}^{2}$]
2. $|ℑ\left(K_{\mathrm{in}\;\mathrm{in}}\right)\;K_{f}\left|\gg \right|K_{\mathrm{out}\;\mathrm{in}}{|}^{2}$            [$3.16\times 10^{9}\;$N${}^{2}\;/$m${}^{4}\gg 2.14\times 10^{8}\;$N${}^{2}\;/$m${}^{4}$]
3. $|K_{f}|\gg \pi^{2}f^{2}M$                                     [$5.85\times 10^{5}\;$N/m${}^{2}\gg 1.20\times 10^{4}\;$N/m${}^{2}$]
4. $ℜ\left(K_{\mathrm{out}\;\mathrm{in}}\right)\gg ℑ\left(K_{\mathrm{out}\;\mathrm{in}}\right)$                  [$1.36\times 10^{4}\;$N/m${}^{2}\gg 5.39\times 10^{3}\;$N/m${}^{2}$]
5. $ℑ\left(K_{\mathrm{in}\;\mathrm{in}}\right)/ℜ\left(K_{\mathrm{out}\;\mathrm{in}}\right)\approx \eta$                [0.396≈0.397]

As a result, the maximum total energy flow transmissibility spectral density from Equation (31) is possible to be approximated as

(32) $${\mathrm{EFT}}_{\max\;\mathrm{uncoupled}}^{\mathrm{appr}}\approx \frac{1}{\eta^{2}}=6.34$$

and is also close to the maximum total energy flow transmissibility spectral density into the foundation ${\mathrm{EFT}}_{\max\;\mathrm{uncoupled}}=6.59$. This surprisingly simple formula implies that the minimum value of the maximum of the total energy flow transmissibility spectral density ${\mathrm{EFT}}_{\max\;\mathrm{uncoupled}}^{\mathrm{appr}}$ is attained by maximizing the loss factor at that frequency. This outcome explains the result of the optimization process for the vibration isolation system studied in this paper—namely, a frequency for the maximum loss factor that is close to the frequency for the maximum total energy flow transmissibility spectral density into the foundation. It also explains that the match is not exact, since the approximations made (1.–5.) are not fully met. In addition, the studied system is not uncoupled. This is studied next.

The contribution of the off-diagonal elements of the compliance matrix H in Equation (9), relative to the diagonal elements, reads $\prod (k_{f}{\;}_{ⓘ}\Delta_{ⓚ})$, where ${}_{ⓘ}\Delta_{ⓚ}$ (i,k=1,2,3,4, i≠k) is the distance between the connections points to the foundation for the vibration isolator bushings ⓘ and ⓚ, respectively. It shows $\lim_{x\to 0|\infty }\prod \left(x\right)=1|0$, respectively. In particular, the extremes $|\prod (k_{f}\left[W-2r_{\mathrm{in}}\right])|=1.00$ at f=0.01 Hz and $|\prod (k_{f}\sqrt{{\left[W-2r_{\mathrm{in}}\right]}^{2}+{\left[L-2r_{\mathrm{in}}\right]}^{2}})|=0.468$ at f=100 Hz. That is, the off-diagonal elements are not small for the studied vibration isolation system, within the considered frequency range. Physically, the relative wavelength extremes $\lambda /(W-2r_{\mathrm{in}})=621$ at f=0.01 Hz and $\lambda /\sqrt{{\left(W-2r_{\mathrm{in}}\right)}^{2}+{\left(L-2r_{\mathrm{in}}\right)}^{2}}=2.04$ at f=100 Hz. That is, the wavelength is not small compared to the distances between the connection points to the foundation for the vibration isolator bushings, within the considered frequency range. In fact, the connection points to the foundation for the vibration isolator bushings are strongly coupled for the studied vibration isolation system, throughout the considered frequency range.

The coupled and uncoupled total energy flow transmissibility spectral densities into the foundation are similar in Figure 7b. However, the total energy in-flow transmissibility spectral density peak is substantially higher for the uncoupled system ${\mathrm{EFT}}_{\mathrm{in}\;\max\;\mathrm{uncoupled}}=104$, as compared to the corresponding peak for the coupled system ${\mathrm{EFT}}_{\mathrm{in}\;\max}=25.5$. The main reason is that the foundation for the uncoupled system is substantially stiffer than for the coupled system, as seen from the mechanical source side in Figure 1—being about four times stiffer in the low-frequency range than for the corresponding coupled vibration isolation system. This stiffness increase results in a higher portion of the energy flow into the vibration isolator bushings is absorbed in the bushings and transformed into heat. In theory, the total energy in-flow transmissibility spectral density peak goes to infinity as the foundation stiffness goes to infinity.

### 3.7. Comparison with a Natural Rubber Vibration Isolation System

The studied hydrogel vibration isolation system is compared to a more traditional vibration isolation system, for the excitation force at [0 0]. To this end, natural rubber vibration isolators are selected, using sulfur cured, unfilled Standard Malaysian Rubber with ingredients and material processing methods given in Kari et al. [49]. Since this material displays a considerably higher storage modulus than the studied hydrogel, it is possible to assume the dynamic stiffness to be

(33) $$K_{\mathrm{in}\;\mathrm{in}\;(m_{\mathrm{in}}=0)}^{\mathrm{NR}}=K_{\mathrm{out}\;\mathrm{out}\;(m_{\mathrm{out}}=0)}^{\mathrm{NR}}=K_{\mathrm{out}\;\mathrm{in}}^{\mathrm{NR}}=\kappa \mu_{\mathrm{st}}^{\mathrm{NR}}\left[1+\frac{\Delta^{\mathrm{NR}}{\left(i\frac{f}{f_{\max[ℑ\left(\mu^{\mathrm{NR}}\right)]}}\right)}^{\alpha }}{1+{\left(i\frac{f}{f_{\max[ℑ\left(\mu^{\mathrm{NR}}\right)]}}\right)}^{\alpha }}\right],$$

where the static modulus $\mu_{\mathrm{st}}^{\mathrm{NR}}=8.25\times 10^{5}\;$N/m${}^{2}$, the relaxation intensity $\Delta^{\mathrm{NR}}=276$, the fractional exponent α=0.657 and the glass transition frequency $f_{\max[ℑ\left(\mu^{\mathrm{NR}}\right)]}=5.41\times 10^{7}\;$Hz. The reader is referred to Kari et al. [49] for the details regarding the experiments and the material properties determination. The dynamic stiffness expression (33) assumes that the wave effects within the natural rubber vibration isolators are negligible within the considered frequency range and that the phase of the dynamic stiffness follows the corresponding phase for the shear modulus. That is, $∠K^{\mathrm{NR}}=∠\mu^{\mathrm{NR}}$. This is a plausible assumption as the absolute value of the shear wavelength $|\lambda^{\mathrm{NR}}|=\sqrt{\mu^{\mathrm{NR}}/\rho^{\mathrm{NR}}}/f=0.293\;$m at f=100 Hz, where $\rho^{\mathrm{NR}}=984\;$kg/m${}^{3}$ from Kari et al. [49]. That is, the wavelength is considerably larger than a typical length of the vibration isolators where shearing takes place—for example, being $r_{\mathrm{out}}-r_{\mathrm{in}}=0.005\;$m for the considered hydrogel vibration isolators. The scaling factor κ is determined by the same constrained non-linear multi-variable programming solver fmincon from Matlab${}^{®}$ [134], to result in the same frequency for the maximum total energy flow transmissibility spectral density into the foundation, as for the hydrogel vibration isolation system, $f_{\max}^{\mathrm{NR}}=f_{\max}$. The resulting scaling factor $\kappa =1.43\times 10^{-2}\;$m.

**Figure 8:**
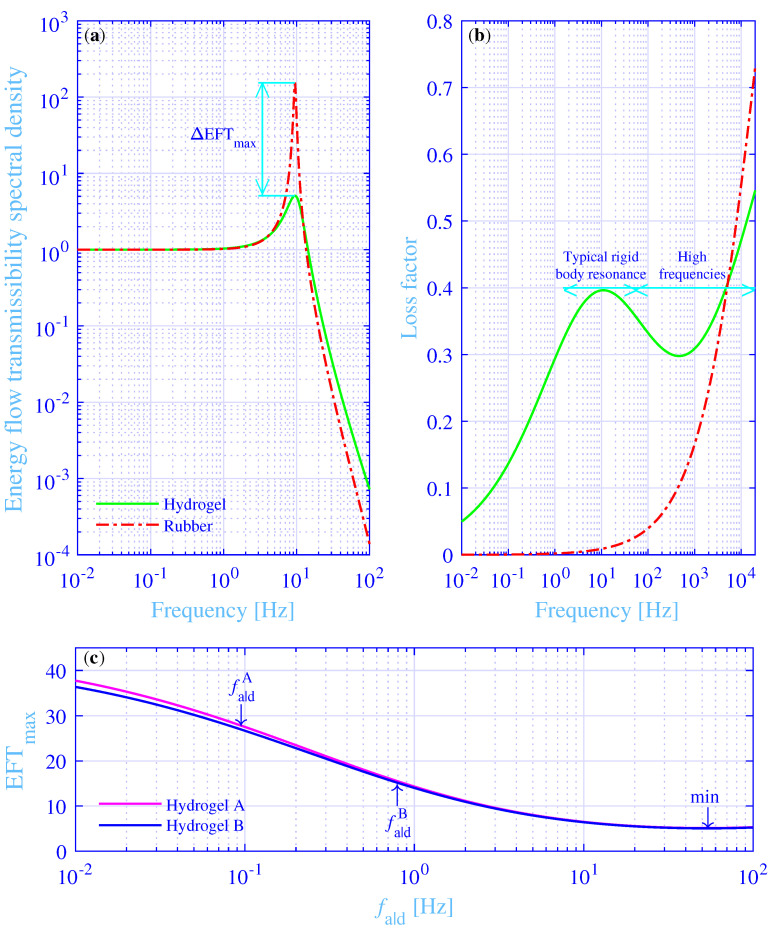
(**a**) The total energy flow transmissibility spectral densities versus the frequency, for the hydrogel vibration isolation system and the more traditional, natural rubber vibration isolation system. (**b**) The loss factors for the tough, single network hydrogel, with both chemical and physical cross-links and for the natural rubber, versus an extended frequency range covering thoroughly the audible frequency range. (**c**) The maximum total energy flow transmissibility spectral densities versus the frequency for the maximum physical loss modulus, for the singe network, doubly cross-linked polyvinyl alcohol hydrogels A and B.

The total energy flow transmissibility spectral density into the foundation versus the frequency, in the range 0.01 to 100 Hz, is shown in Figure 8a, for the excitation force at [0 0]. The total energy flow transmissibility spectral density, for the hydrogel vibration isolation system, is shown in a green solid line, while the corresponding total energy flow transmissibility spectral density, for the traditional vibration isolation system, is shown in a red dash–dotted line. Clearly, the traditional vibration isolation system shows an essentially higher peak value ${\mathrm{EFT}}_{\max}^{\mathrm{NR}}=154$, at $f_{\max}^{\mathrm{NR}}=9.49\;$Hz, as compared to the peak ${\mathrm{EFT}}_{\max}=5.09$ for the hydrogel vibration isolation system, at the same frequency $f_{\max}=9.49\;$Hz. In fact, their ratio $\Delta {\mathrm{EFT}}_{\max}={\mathrm{EFT}}_{\max}^{\mathrm{NR}}/{\mathrm{EFT}}_{\max}=30.2$—that is, it is more than 30 times higher. The total energy flow transmissibility spectral density is only slightly higher for the hydrogel in the high-frequency end of Figure 8a and is due to a higher loss factor, as compared to the corresponding for the traditional vibration isolation system. This is considered next.

The loss factors for the hydrogel and the traditional vibration isolator materials are shown in Figure 8b for an extended frequency range, from 0.01 Hz to 20,000 Hz—thus, well covering the audible frequency range. The loss factor, for the hydrogel vibration isolator material, is shown in a green solid line while the corresponding loss factor, for the traditional vibration isolator material, is shown in a red dash–dotted line. Clearly, the hydrogel vibration isolator material shows a high loss factor throughout the typical frequency range for rigid body resonances of vibration isolation systems, while the corresponding traditional vibration isolator material shows a surprisingly low loss factor, within the same frequency range. The prodigious loss factor difference explains the large variation obtained for the total energy flow transmissibility spectral densities into the foundation. On the contrary, the hydrogel vibration isolator material shows a moderate to non-vanishing loss factor in the high frequency range, where some damping is needed to decrease the total energy flow transmissibility spectral density peaks into the foundation due to possible internal anti-resonances, within the vibration isolators that most likely will occur within the audible frequency range. On the other hand, the damping is not supposed to be overly high since that generally will increase the total energy flow transmissibility spectral densities. This is exactly what is happening in the high-frequency range end for the traditional vibration isolator material in Figure 8b, where the loss factor is 0.50 and more. It should be noted that the extrapolation of the hydrogel loss factor into the high-frequency part of the audible frequency range in Figure 8b, should be interpreted with caution since the stress–strain model in Kari [33] is experimentally validated up to 100 rad/s—that is, up to 16 Hz. However, the hydrogel stress–strain model is experimentally validated at the rigid body frequency range of the vibration isolation system. Likewise, the stress–strain model for the natural rubber is experimentally validated throughout the considered frequency range [49].

Finally, two special cases of singe network, doubly cross-linked polyvinyl alcohol hydrogels vibration isolation systems are studied, for the excitation force at [0 0]. Namely, the materials with the measurement results fitted to the constitutive model developed in Kari [33] and with the measurement and material details from Mayumi et al. [12] (Hydrogel A) and Zhao et al. [29] (Hydrogel B). Chemical cross-link densities are supposed to be scalable to result in an equivalent static modulus as in this study—namely, $\mu_{\mathrm{st}}=2000\;$N/m${}^{2}$. Moreover, the maximally active physical-to-chemical cross-link density ratios are supposed to be scalable to result in an equivalent relaxation intensity as in this study—namely, Δ=5. Furthermore, the hydrogel densities are supposed to be the same as in this study, ρ=1000 kg/m${}^{3}$. Lastly, the chemical part of hydrogel shear modulus in Equation (5) applies the chemical Rouse stress intensity factors $C^{A}=0.0628\sqrt{s}$ and $C^{B}=0.0696\sqrt{s}$ [5], while the frequencies for the maximum physical loss modulus are fixed to $f_{a|d}^{A}=0.6/2\pi \;$Hz and $f_{a|d}^{B}=5.0/2\pi \;$Hz [33]. The maximum total energy flow transmissibility spectral densities into the foundation versus the frequency for the maximum physical loss modulus, in the extended range 0.01 to 100 Hz, are shown in Figure 8c, where the frequency for the maximum physical loss modulus is admitted to vary. The result for the hydrogel A is in a magenta solid line while the corresponding result for the hydrogel B is in a blue solid line. The fixed values of the frequencies for the maximum physical loss modulus are marked with arrows. Clearly, the hydrogel A and B lines are almost overlapping, with hydrogel A showing a slightly higher value throughout the whole considered frequency range. They start in the low-frequency limit, at $f_{a|d}=0.01\;$Hz, from just below 40. Then they decrease with increasing frequency to minimum values $\min[{\mathrm{EFT}}_{\max}^{A}]=5.12$ at $f_{a|d}^{A\;\mathrm{opt}}=52.3\;$Hz and $\min[{\mathrm{EFT}}_{\max}^{B}]=5.05$ at $f_{a|d}^{B\;\mathrm{opt}}=53.1\;$Hz, respectively. Finally, they increase slightly with increasing frequency above those minima. The corresponding minimum $\min[{\mathrm{EFT}}_{\max}]=5.09$ at $f_{a|d}^{\mathrm{opt}}=52.7\;$Hz for the excitation force at [0 0] (from the Table 2) lies in between those minima. Not surprisingly, as the chemical Rouse stress intensity factor C is the average of hydrogels A and B and lies in between those factors $C^{A}<C<C^{B}$. Finally, the maximum total energy flow transmissibility spectral densities into the foundation read ${\mathrm{EFT}}_{\max}^{A}=27.8$, at the frequency for maximum physical loss modulus $f_{a|d}=f_{a|d}^{A}=0.6/2\pi \;$Hz and ${\mathrm{EFT}}_{\max}^{B}=15.2$ at $f_{a|d}=f_{a|d}^{B}=5.0/2\pi \;$Hz. The resulting ratios read $\Delta {\mathrm{EFT}}_{\max}^{A}={\mathrm{EFT}}_{\max}^{\mathrm{NR}}/{\mathrm{EFT}}_{\max}^{A}=5.55$ and $\Delta {\mathrm{EFT}}_{\max}^{B}={\mathrm{EFT}}_{\max}^{\mathrm{NR}}/{\mathrm{EFT}}_{\max}^{B}=10.2$, respectively. Surprisingly, the hydrogels A and B show, as such and without any optimization of the frequency for the maximum physical loss modulus, a clear advantage in reducing the maximum of the total energy flow transmissibility spectral density into the foundation, as compared to the traditional vibration isolation materials, such as natural rubber. Clearly, it is an advantage for the polyvinyl alcohol hydrogels that are missing, to a moderate extent, the possibility to vary the kinetics and thermodynamics of the adhesion–deadhesion activities of the physical borate esterification cross-links in order to alter the frequency for the maximum physical loss modulus [24].

## 4. Conclusions

A simulation model is developed for the energy flow transmissibility from the machine source and into the receiving foundation for a realistic vibration isolation system, displaying several rigid body resonances and various energy flow transmission paths. It shows that a significant peak energy flow transmissibility reduction is possible while using materials typically applied in tissue engineering—namely, tough, doubly cross-linked, single polymer network hydrogels, with both chemical and physical cross-links, as vibration isolator material, instead of more traditional materials, such as natural rubber. The physical reason to the substantial peak reduction is the intensive adhesion–deadhesion activities of the physical cross-links, resulting in a high loss factor. This loss factor peak is possible to set close to the energy flow peaks for the vibration isolation system. Moreover, it is shown that a considerable reduction is also possible without any optimization of the loss factor peak position—a clear advantage for polyvinyl alcohol hydrogels that are somewhat missing the possibility to alter the frequency for the maximum physical loss [24]. An interesting continuation of the work performed is to investigate the practical aspects of the tough hydrogel, multi-degree-of-freedom vibration isolation systems, including their durability, aging, economical aspects and a thorough energy flow measurement. Those aspects involve a great deal of work and are beyond the scope of the present paper.

## Figures and Tables

**Table 1:** The dimensions, the mechanical and material parameter quantities and values for the mechanical source, vibration isolator bushings and for the foundation.

Mechanical Source			Vibration Isolators			Foundation		
Quantity		Value	Quantity		Value	Quantity		Value
Width	*W*	0.200 m	Length	*l*	0.100 m	Young’s		
Length	*L*	0.500 m	Inner diameter	$d_{\mathrm{in}}$	0.040 m	modulus	$\Upsilon_{f}$	210 GN/m${}^{2}$
Hight	*H*	0.050 m	Outer diameter	$d_{\mathrm{out}}$	0.050 m	Poisson’s		
Density	$\rho_{M}$	2700 kg/m${}^{3}$	Inner sleeve mass	$m_{\mathrm{in}}$	0.407 kg	ratio:	$\nu_{f}$	0.300
Mass	*M*	13.50 kg	Outer sleeve mass	$m_{\mathrm{out}}$	0.111 kg	Thickness	*h*	0.010 m
Moment			Density	ρ	1000 kg/m${}^{3}$	Density	$\rho_{f}$	780 kg/m${}^{2}$
of inertia	$J_{x}$	0.284 kg m${}^{2}$	Static modulus	$\mu_{\mathrm{st}}$	2000 N/m${}^{2}$		
Moment			Relaxation intensity	Δ	5.00		
of inertia	$J_{y}$	0.478 kg m${}^{2}$	Chemical Rouse stress intensity factor				C	0.0662 $\sqrt{s}$
			Max physical loss modulus frequency			$f_{a|d}=\omega_{a|d}/2\pi$		50 to 53 Hz

**Table 2:** The optimized frequencies for the maximum physical loss factor $f_{a|d}^{\mathrm{opt}}$ in green, with resulting hydrogel vibration isolation system parameters, for all the nine combinations of the force excitation points [n m], with n,m=0,1,2. The corresponding frequency $f_{\max}$ for the minimum of the maximum energy flow transmissibility spectral density $\min[{\mathrm{EFT}}_{\max}]$ and the frequency $f_{\mathrm{in}\;\max}$ for the maximum energy in-flow transmissibility spectral density ${\mathrm{EFT}}_{\mathrm{in}\;\max}$. The frequency $f_{\max[\eta ]}$ for the maximum total loss factor and the frequency $f_{\max[\eta_{\mathrm{phys}}]}$ for the maximum physical loss factor. The corresponding frequency for the maximum physical loss factor using C=0 is in parenthesis.

[n m]	$f_{a|d}^{\mathrm{opt}}\;$[Hz]	$\min[{\mathrm{EFT}}_{\max}]$	$f_{\max}\;$[Hz]	${\mathrm{EFT}}_{\mathrm{in}\;\max}$	$f_{\mathrm{in}\;\max}\;$[Hz]	$f_{\max[\eta ]}\;$[Hz]	$f_{\max[\eta_{\mathrm{phys}}]}\;$[Hz]
[0 0]	52.7	5.09	9.49	25.5	9.75	11.0	7.81 (8.78)
[0 1]	52.2	5.02	9.50	27.7	9.88	10.9	7.75 (8.70)
[0 2]	50.9	4.83	9.55	44.5	15.1	10.6	7.57 (8.49)
[1 0]	52.7	5.08	9.49	29.6	9.99	11.0	7.81 (8.78)
[1 1]	52.2	5.01	9.51	31.8	10.2	10.9	7.74 (8.70)
[1 2]	50.9	4.83	9.55	51.0	14.7	10.6	7.57 (8.49)
[2 0]	52.6	5.06	9.50	50.7	12.8	11.0	7.80 (8.77)
[2 1]	52.1	5.00	9.51	56.4	13.1	10.9	7.74 (8.69)
[2 2]	50.9	4.82	9.56	73.7	13.9	10.6	7.56 (8.48)

## Data Availability

Data is contained within the article.
